# Bruton’s tyrosine kinase inhibition re-sensitizes multidrug-resistant DLBCL tumors driven by BCL10 gain-of-function mutants to venetoclax

**DOI:** 10.1038/s41408-025-01214-y

**Published:** 2025-02-02

**Authors:** Caroline A. Coughlin, Dhanvantri Chahar, Marianna Lekakis, Abdessamad A. Youssfi, Lingxiao Li, Evan Roberts, Natalia Campos Gallego, Claude-Henry Volmar, Ola Landgren, Shaun Brothers, Anthony J. Griswold, Catalina Amador, Daniel Bilbao, Francesco Maura, Jonathan H. Schatz

**Affiliations:** 1 University of Miami Miller School of Medicine Medical Scientist Training Program, Miami, Fl USA; 2 Division of Hematology, Department of Medicine, University of Miami Miller School of Medicine, Miami, Fl USA; 3 Sylvester Comprehensive Cancer Center, Miami, FL USA; 4 GenScript ProBio, Nanjing, China; 5 Center for Therapeutic Innovation, University of Miami, Miami, FL USA; 6 Department of Psychiatry and Behavioral Sciences, University of Miami, Miami, FL USA; 7 Division of Myeloma, Department of Medicine, University of Miami School of Medicine, Miami, Fl USA; 8 John P. Hussman Institute for Human Genomics, University of Miami Miller School of Medicine, Miami, FL USA; 9 Department of Pathology and Laboratory Medicine, University of Miami Miller School of Medicine, Miami, FL USA

**Keywords:** B-cell lymphoma, Cancer therapeutic resistance

## Abstract

Disparate pathogenic mechanisms complicate precision-medicine efforts to treat diffuse large B-cell lymphoma (DLBCL), the most common lymphoma diagnosis. Though potentially curable with frontline combination chemoimmunotherapy, DLBCL carries persistently poor prognosis for those with relapsed or refractory (rel/ref) disease, despite recent advances in immunotherapy. Here, we build on recent findings implicating gain-of-function mutations in the BCL10 signaling protein as drivers of resistance to Bruton’s tyrosine kinase (BTK) inhibitors. We show mutant BCL10-driven DLBCL is resistant to multiple additional drug classes, demonstrating urgency to derive mechanistically rooted strategies to overcome undruggable BCL10 mutants that stabilize BTK-independent signaling filaments upstream of NF-kB activation. BCL10 mutants promote a cytokine-reinforced positive feedback loop of lymphomagenesis driving not just NF-kB but multiple additional pathways converging on diffuse activation of oncogenic transcription factors. Up-regulation of anti-apoptotic genes increases mitochondrial membrane potential, underlying multidrug resistance. Increased expression of *BCL2*, *BCL2L1* (*BCL-XL*), and *BCL2A1* (*BFL1*) drives resistance to venetoclax, but expression can be overcome by the potent non-covalent BTK inhibitor pirtobrutinib. Venetoclax plus pirtobrutinib synergized in overcoming resistance and potently killed BCL10-mutant lymphomas in vitro and in vivo. BTK therefore retains key roles protecting DLBCL from apoptosis even when downstream activation of the BCL10 signaling complex activates NF-kB independently.

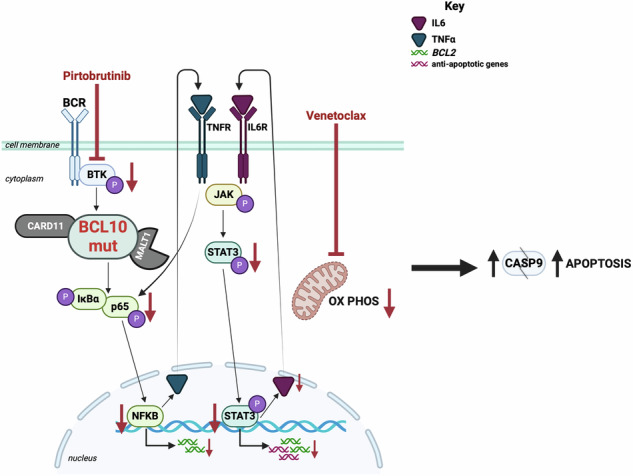

## Introduction

Signaling downstream from the B-cell receptor (BCR) drives diffuse large B-cell lymphoma (DLBCL) progression [1, 2]. Activated B-cell (ABC) cell of origin (COO)-derived tumors in particular require activation of NF-kB by the CARD11-BCL10-MALT1 (CBM) complex that integrates signals from the BCR and other cell-surface molecules. Numerous genomic alterations converge on CBM activation in DLBCL, including mutations of *CD79B*, *MYD88*, *CARD11*, and *TNFAIP3* [3, 4]. Heterogeneous mutational and signaling profiles are key factors thwarting clinical activities of targeted signaling inhibitors that had no role in DLBCL standard frontline or curative-intent salvage therapy until the recently published ViPOR salvage regimen (see discussion) [5]. Identification of rational combinations linked to specific biomarkers is a key priority for DLBCL patients failed by frontline chemoimmunotherapy, who continue to have poor prognosis despite recent treatment advances [6, 7].

Recently, *BCL10* gain-of-function mutations emerged as additional drivers of CBM activation in DLBCL. These include a recurrent R58Q CARD-domain substitution that stabilizes CBM complexes and truncations of BCL10’s ST-rich C-terminal the promote spontaneous formation of activated BCL10 signaling filaments [8]. Found overall in ~5% of newly diagnosed DLBCL, BCL10 alterations cluster strongly in ABC-enriched LymphGen BN2/Chapuy C1 cases characterized by *BCL6* translocations and *NOTCH2* mutations [9–11]. These cases specifically experience no benefit from addition of the covalent BTK inhibitor (BTKi) ibrutinib to frontline therapy regardless of patient age, while other groups enriched in ABC cases had improved outcomes in patients under 60 [12]. Correspondingly, *BCL10* mutations, functioning downstream from BTK in BCR signaling, were specifically shown to mediate BTKi resistance [8]. As new BTKis are developed, including the noncovalent inhibitor pirtobrutinib, further evaluation of key signaling pathways in relation to BCL10 mutations is necessary to understand potential therapeutic benefits for this subgroup of DLBCL.

MALT1 paracaspase activity is a key output of CBM activation, cleaving negative regulators of NF-kB signaling like *TNFAIP3*-encoded A20 and fine-tuning gene expression though cleavage of Regnase-1 and Roquin-1/2 [13]. MALT1 paracaspase inhibitors have entered clinical evaluation, and importantly BCL10 mutant-driven cells retain sensitivity to these compounds [8]. Paracaspase inhibition, however, does not impact MALT1’s crucial scaffolding roles in orchestrating the downstream events that activate NF-kB. Further consequences of BCL10 mutant-mediated CBM activation remain undefined.

Here, to address treatment resistance in BN2 cases, we assessed the impact of *BCL10* mutations. Gene-expression profiling revealed extensive reprogramming of cytokine production and signaling. Compound library screening revealed multiple drug classes thwarted by mutant-BCL10 proteins. These include the BCL2 inhibitor venetoclax, an FDA-approved drug with remarkable activities in some other B-cell non-Hodgkin lymphomas. We find that though CBM activation promotes BTKi resistance, cells with *BCL10* mutations nonetheless remain dependent on the enzyme to provide protection from apoptosis and maintain venetoclax resistance.

## Materials and methods

### Cell lines and reagents

Cell lines came from ATCC and collaborators and were verified for authenticity. Sources of compounds utilized in cell-based experiments are in Supplemental Table 1.

### Cloning and overexpression of BCL10 mutants

The QuikChange II XL Site-Directed Mutagenesis Kit (Agilent) generated *BCL10* mutants in the plvx Tetone vector. Virus was generated by transfecting 293 T cells with plvx-IRES-ZsGreen1 or pLVX-Tetone-puro. Infection efficiency was measured with GFP expression and selected with puromycin.

### Competition assay

Cells were infected with plvx-IRES-ZsGreen1 vectors containing empty vector, BCL10 wild type and mutants as described above. GFP was measured using an Attune cytometer. Cells were treated with BTKis for 72–96 h and then washed out of drug and allowed to recover. This step was repeated twice and GFP was measured every 48 h.

### Cell viability and synergy

Cells were seeded at 5000/well in 96-well plates or 1000/well in 384-well plates in serial drug dilutions. Viability was assessed by Cell Titer Glo (Promega). Luminescence was measured, and nonlinear fit regression analysis in GraphPad Prism determined EC_50_. Bliss synergy score was calculated on: http://synergyfinder.fimm.fi/synergy/20210604233713824028/

### Dual Luciferase reporter

2.5 × 10^5^ 293 T cells we plated in 12-well plates and transfected 24 h later with 50 ng NFkB, 25 ng Renilla and 50 ng plasmid of interest using Lipofectamine. Cells were incubated 40 h with readout by Dual Luciferase Reporter Assay kit (Promega).

### EGFP NF-kB reporter

The pHAGE-6x-NF-kB-conA-HygEGFP plasmid was a gift from D. Krappmann (Helmholtz Zentrum), used as previously described [14]. Infected cells were treated with 200 ng/ml of doxycycline for 24 h. Flow cytometry measured GFP as NF-kB readout compared to uninduced cells.

### Western blot

Cells were lysed in RIPA (ThermoScientific) and protein quantified using Bradford reagent (Biorad). Equal amounts of proteins were resolved on SDS-PAGE and transferred to PVDF membranes (Biorad). Membranes were blocked with 5% milk and probed with primary antibodies (Supplemental Table 2) followed by host-specific secondary antibodies with chemiluminescence measured by LiCor imaging (LI-COR,NE, USA).

### Enzyme linked immunosorbent assay (ELISA)

1 × 10^6^ cells were cultured with supernatant collected to measure cytokines by DuoSet ELISA (R&D Systems) according to manufacturer’s protocol.

### Phospho-Kinase and cytokine arrays

Human Phospho-Kinase Array (R&D Systems, #ARY003C) and Human XL Cytokine Array (R&D Systems: ARY022B) were performed by manufacturer’s protocol using lysates and supernatant from doxycycline-induced RIVA cells.

### RNA sequencing and analyses

RIVA cells infected with indicated plvx-Tetone vectors were induced with 200 ng/ml of doxycycline x24 h. RNA was extracted (Qiagen RNAeasy Mini Kit with optional on-column DNAse) and sequenced on Illumina NovaSeq 6000. The samples were polyA selected and underwent 30 M SE100 reads. Transcription-factor enrichment analysis employed ChIP-X Enrichment Analysis Version 3 (ChEA3) [15]. Gene Set Enrichment Analysis (GSEA) employed version 4.3.2. Gene Ontology (GO) analysis employed https://maayanlab.cloud/Enrichr/ [16].

### Drug screens

Drug screens employed the Epigenetic Library (TargetMol): https://www.targetmol.com/compound-library/Epigenetics_Compound_Library.

### In vivo experiments

All mouse studies were approved by the University of Miami Institutional Animal Care and Use committee and were in compliance with ethical regulations. Mice were housed in pathogen-free conditions. Cages are handled under animal transfer stations (Biological Safety Cabinets). Mice are housed in individually ventilated, double sided, racks changed weekly. The investigators were not blinded to the experimental groups. 12-weeks old female mice were randomly assigned to the four experimental groups: Control (*n* = 8), pirtobrutinib (*n* = 8), venetoclax (*n* = 8) and venetoclax+pirtobrutinib combination (*n* = 8). At the endpoints mice were euthanized and tumors were collected. Pirtobrutinib for in vivo was obtained at pharmacologic grade from Eli Lilly, Inc. Cell line xenografts started with 2 × 10^6^ cells flank injected to NOD scid gamma mice (The Jackson Laboratory).

### Mitochondrial membrane potential

Cells were collected and incubated with 200 nM Tetramethylrhodamine methyl ester (TMRM) as described [17], with results acquired on an Attune NxT Cytometer (Thermo Fisher Scientific, USA).

### Quantitative real-time PCR

Total RNA (Allprep DNA/RNA Mini kit, Qiagen) underwent SuperScript™ III Reverse Transcription (Thermoscientific). Quantitative PCR used PowerTrack™ SYBR Green Master Mix (Thermoscientific) with expression determined by Steponeplus (Applied Biosystems, Thermo Fisher Scientific), normalized to 18 s rRNA as described [18]. Primers (Supplemental Table 3) were by Primer 3.0 software or in published literature [19–21].

### Histology and immunohistochemistry

Tumors were fixed in 10% formalin. Sample preparation, processing, H&E staining were as described [22]. All staining used a Bond RX automated stainer (Leica Microsystems, Wetzlar, Germany) and images were captured on an Olympus BX43 microscope (Olympus, Tokyo, Japan).

### Quantification and statistical analysis

Experiments were performed in three independent replicates unless otherwise noted. Statistical analyses employed GraphPad Prism version 9. Unpaired student’s *t* tests assessed statistical significance: ns, *p* > 0.05; *p ≤ 0.05; **p ≤ 0.01; ***p ≤ 0.001 and ****p ≤ 0.0001). For animal study number of animals assigned per group were based on the ≥85% power to detect an effect size of 1.6 SDs between groups at two-sided significance of 5%.

## Results

### Gene-expression impact of BCL10 mutations

To assess mechanisms of BCL10-mutant lymphomagenesis, we established gain-of-function systems in ABC-DLBCL cell lines. Both classes of mutation strongly activate an NF-kB reporter in 293 T cells with or without phorbol myristate acetate (PMA)/ionomycin (P/I) stimulation (Supplemental Fig. 1A). We focused moving forward on R58Q that stabilizes formed CBM complexes [8] and the truncation S136X. Introduction in tetracycline-inducible (tet-on) incorporating lentiviral constructs to *BCL10* CRISPR-deleted JURKAT cells demonstrated reliable induction with minimal leaky expression (Supplemental Fig. 1B). RIVA ABC-DLBCL cells also induced expression reliably within four hours, reaching stable maximum by 16–24 h (Supplemental Fig. 1C, D). We similarly engineered the ABC-DLBCL lines HBL1, U2932, and TMD8. Induced expression of WT or mutant BCL10 did not provide proliferative advantage (Supplementary Fig. 1E) in these lines that constitutively activate CBM in an obligate manner [4]. These systems therefore permit interrogation of BCL10-mutant mechanisms independently of cell proliferation effects. We employed RIVA as primary discovery system due to derivation from BN2/C1 DLBCL with both *BCL6* rearrangement and *NOTCH2* activating mutation [9, 11].

**Fig. 1:**
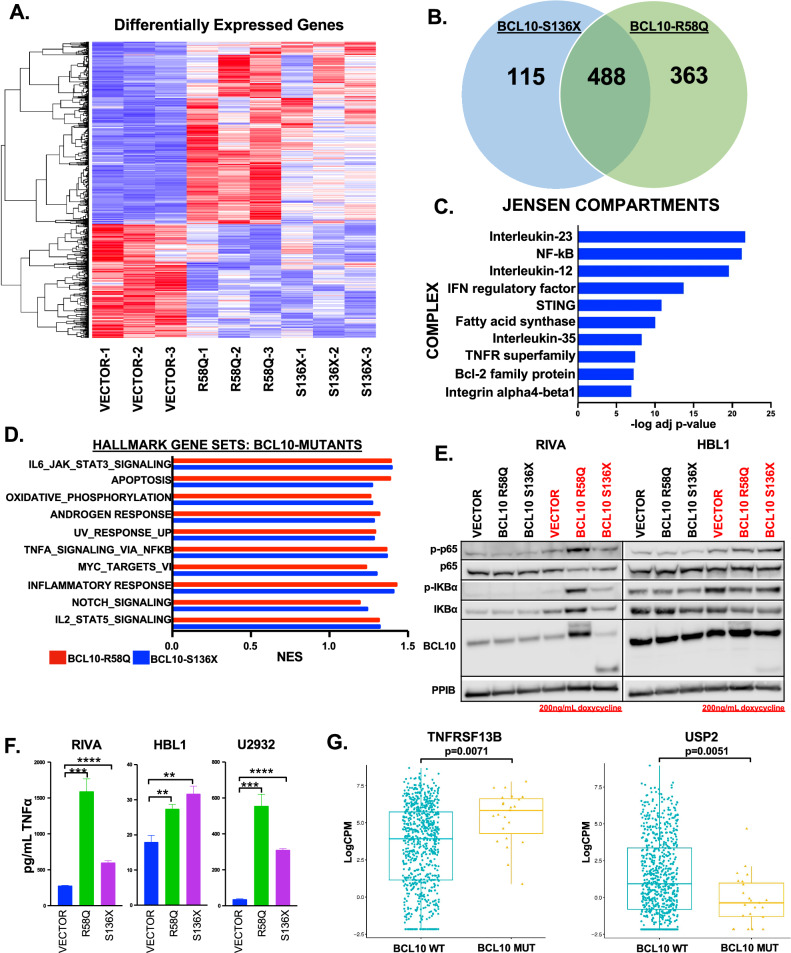
Gene expression impact of BCL10 mutations. **A** Heatmap of differentially expressed genes in RIVA cells containing vector or BCL10 mutants, (FC ≥ 1.5x up or down)**. B** Venn diagram of the number of differentially expressed genes in BCL10 mutants compared to vector. **C** Top 10 upregulated (adjusted p value ≤ 0.001) Jensen compartment complexes using genes upregulated with a FC ≥ 2. **D** The top overlapping Hallmark gene set pathways upregulated (FDR q-value ≤ 0.25) in BCL10 mutants. **E** Western blot of RIVA and HBL1 cells induced with doxycycline for 24 h and probed as indicated. **F** TNFα ELISA assay on cell supernatant from RIVA, HBL1, and U2932 cells containing vector or BCL10 mutants. **G** Boxplot of gene expression levels of *TNFRSF13B* and *USP2* of untreated DLBCL cases comparing mutant versus wildtype BCL10 cases.

Biologic replicates of RIVA cells with S136X, R58Q, or empty vector (Supplemental Fig. 1F) underwent RNA-seq 24 h after addition of 200 ng/mL doxycycline (GEO Accession# GSE267421). Unsupervised clustering by differential expression (≥1.5x up or down) showed clear distinction between vector and *BCL10* mutant-expressing samples (Fig. 1A). The heatmap suggested broad similarity between the two mutants, and indeed comparison of each replicate group separately to vector showed 488 genes differentially expressed in common (Fig. 1B, Supplemental Fig. 2). Jensen compartment analysis of commonly up-regulated genes confirmed NF-kB as a top up-regulated protein complex (Fig. 1C, Supplemental Table 4) [23]. Gene-set enrichment analysis (GSEA) of each mutant group compared to vector showed significant overlap (FDR q-value < 0.25) in deregulated processes (Fig. 1D, Supplemental Table 5), with results broadly similar to the Jensen compartments. Immunoblotting confirmed canonical NF-kB activation (Fig. 1E), and we found significantly increased production of TNFα in supernatants of the mutant-expressing cells (Fig. 1F). Notably, both Jensen and GSEA suggested deregulation of apoptosis including BCL2 family members (see below). We next assessed RNA-seq data from Reddy et al., cohort of untreated DLBCL patients, comparing BCL10-mutant cases (*n* = 25, cBioPortal.org) to all others (*n* = 750) [24]. As expected, the number of BCL10 mutant cases is relatively small (3.2%), limiting multivariate analyses. Despite these limitations, we found deregulation of key TNFα-related genes in BCL10-mutant cases (Fig. 1G). These results reveal broad reprogramming of DLBCL biology by lymphomagenic *BCL10* mutants.

### BCL10 mutants promote production of oncogenic cytokines

**Fig. 2:**
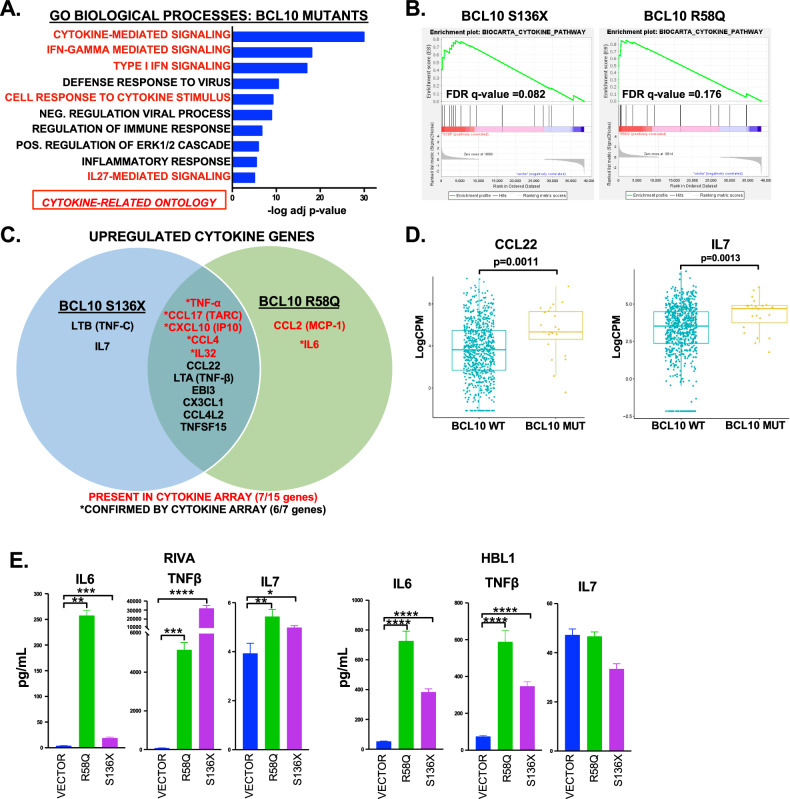
BCL10 mutations promote production of oncogenic cytokines. **A** Gene ontology analysis of genes upregulated in BCL10 mutant RIVA cells showing top 10 of 49 significantly upregulated ontologies (adjusted *p* < 0.001). Ontologies that were completely overlapping in genes were combined. **B** GSEA “BIOCARTA_CYTOKINE_PATHWAY” for BCL10 mutants (q = 0.082 S136X, q = 0.176 for R58Q). **C** Venn diagram of cytokine genes upregulated in BCL10 mutants with array validations indicated. **D** Boxplot of gene expression levels of *CCL22* and *IL7* of untreated DLBCL cases comparing mutant versus wildtype BCL10 cases. **E** ELISA assays of IL6, IL7 and TNFβ on cells supernatant of RIVA and HBL1 cells induced for 24 (IL7 and TNFβ) or 72 h (IL6) with doxycycline, **p < 0.01; ***p < 0.001; ****p < 0.0001.

These results prompted us to investigate cytokines as a unifying mechanism. IL-6 and IL-10 previously were implicated as autocrine factors in ABC-DLBCL [25], but cytokines have not been examined in the mutant-BCL10 context or in an unbiased fashion in ABC-DLBCL. Strikingly, gene-ontology (GO) analysis of differential expression by both mutant classes revealed cytokine-mediated signaling as by far the most upregulated (Fig. 2A, Supplemental Table 6). Indeed, of the top 10 most upregulated ontologies, five were cytokine related. “BIOCARTA_CYTOKINE _PATHWAY” was highly significantly enriched in GSEA for genes deregulated by both mutants (Fig. 2B). We therefore investigated specific cytokines whose increased production might be implicated. Figure 2C shows cytokines at the gene-expression level. IL-10 was not further deregulated compared to baseline, while *IL-6* expression was significant only for R58Q. We next employed cytokine arrays on supernatants 72 h after induction (Supplemental Fig. 3). As indicated, 7/15 cytokines upregulated at the mRNA level were present on the array, and six followed the same pattern as mRNA. Cytokines upregulated by the mutants were also significantly upregulated in BCL10-mutant patient tumors including *CCL22* (*p* = 0.0011) and *IL7* (*p* = 0.0013) (Fig. 2D). Finally, we used ELISA to further confirm specific cytokines independently, showing activation of TNFβ in addition to TNFα (Figs. 1F, 2E). Both mutants consistently increased IL-6 production, with the effect greater downstream of R58Q in line with the RNA-seq result. These findings implicate cytokine production as a mechanism of lymphomagenesis downstream of activating BCL10 mutations with implications for autocrine stimulation and reprogramming of lymphoma microenvironments.

### BCL10 mutants deregulate multiple transcription factors

**Fig. 3:**
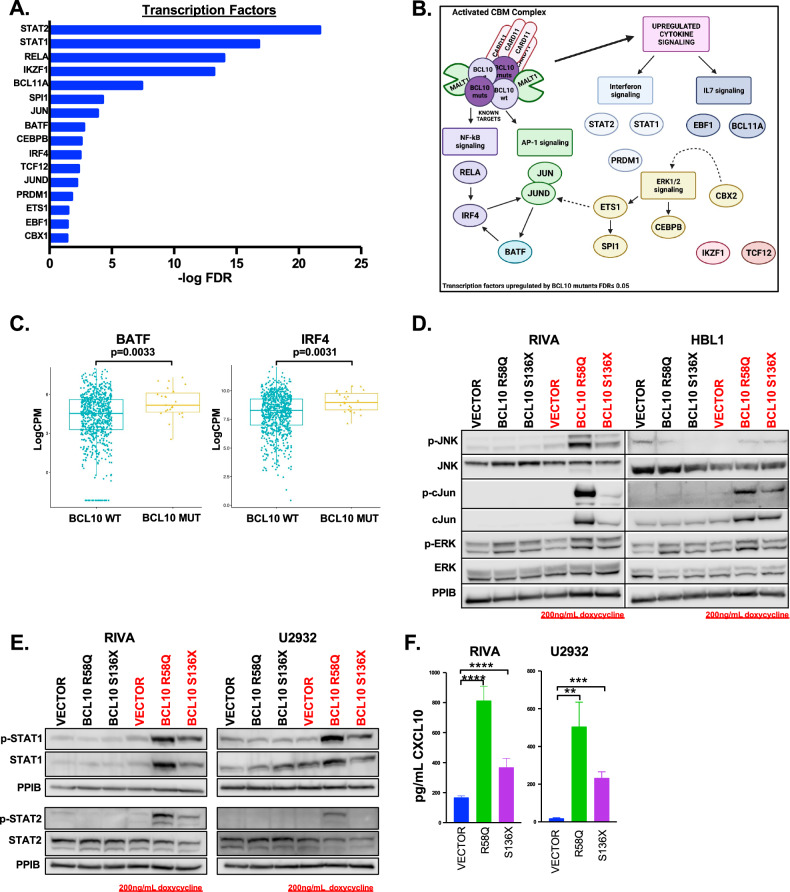
BCL10 mutants mediate lymphomagenesis through activation of key transcription factors. **A** Genes that were upregulated (FC ≥ 2) in both mutants were analyzed for enrichment in regulation by transcription factors from the ENCODE project through the online transcription factor analysis tool ChIP-X Enrichment Analysis Version 3 (ChEA3). Sixteen transcription factors were significantly upregulated in BCL10 mutants (FDR ≤ 0.05). **B** Schematic of transcription factors identified to be upregulated in (**A**). **C** Boxplot of gene expression levels of *BATF* and *IRF4* of untreated DLBCL cases comparing mutant versus wildtype BCL10 cases. **D** Western blot of RIVA and HBL1 cells induced with doxycycline for 24 h and probed as indicated. **E** Western blot of RIVA and U2932 cells induced with doxycycline for 24 h and probed as indicated. **F** ELISA assays in RIVA AND U2932 cell supernatant probing for CXCL10 (RIVA - S136X: *p* = 0.0046, R58Q: *p* = 0.0003, U2932- S136X: *p* = 0.0003, R58Q: *p* = 0.0028).

Next, we assessed the transcription factors promoting gene-expression reprogramming by BCL10 mutants. Genes upregulated (FC ≥ 2) in both mutants were analyzed for enrichment in transcription factors from ENCODE (see methods) [15]. Sixteen transcription factors were implicated (FDR ≤ 0.05, Fig. 3A, Supplemental Table 7). As expected, transcription factors downstream of CBM including NF-kB (*RELA* and *IRF4*) and AP-1 (*JUN* and *JUND*) were present. In addition, ERK1/2-mediated transcription factors *CEBPB*, *ETS1,* and *SPI1* were implicated, along with those mediated by interferons (*STAT1, STAT2, PRDM1*) and IL7 (*EBF1, BCL11A*) (Fig. 3B). Patient data confirmed key findings, including upregulation of *BATF* (*p* = 0.0033) and *IRF4* (*p* = 0.0031) in BCL10-mutant tumors (Fig. 3C). Western Blot also confirmed upregulation of AP-1 signaling indicated by phosphorylated JNK and phosphorylated and total cJun (Fig. 3D). In addition, the gene ontology results highlighted activation of ERK1/2. STAT1 and STAT2 were the most significantly implicated transcription factors by ENCODE and upregulation of their active forms was confirmed by western blot (Fig. 3E). Correspondingly, there was increased production of the IFNγ inducible cytokine, CXCL10 (Fig. 3F). This confirms GO Biological Process results showing IFNγ signaling as the second most upregulated gene ontology in the BCL10 mutant cells (Fig. 2A).

### BCL10 mutations thwart efficacy of multiple drug classes

**Fig. 4:**
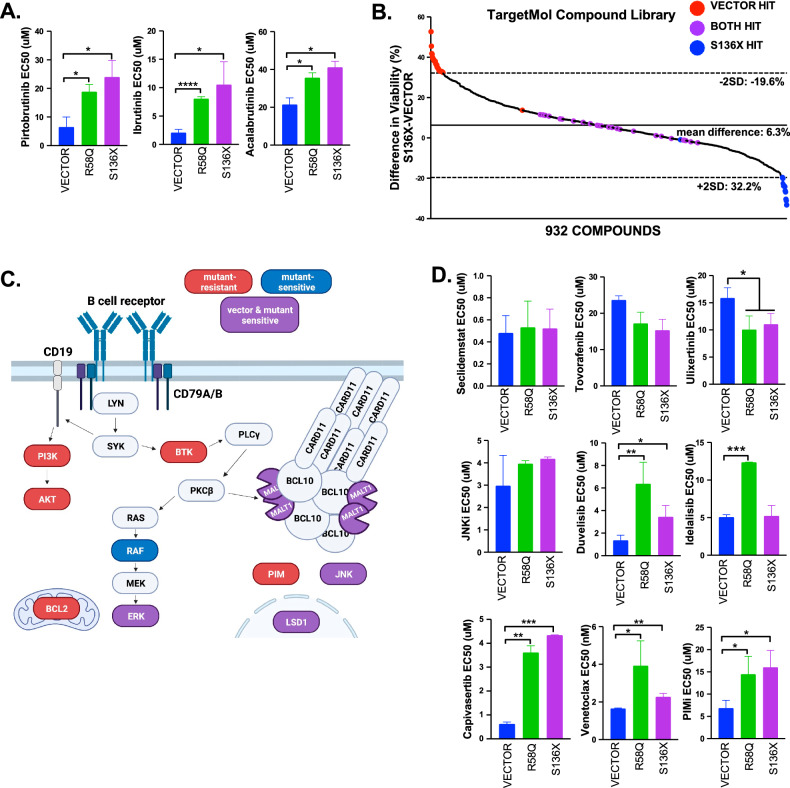
BCL10 mutants drive resistance to multiple drug classes. **A** Dose response viability assays of RIVA cells treated with pirtobrutinib (S136X: *p* = 0.033, R58Q: *p* = 0.019), acalabrutinib (S136X: *p* = 0.07, R58Q: *p* = 0.07), and ibrutinib (S136X: *p* = 0.023, R58Q: *p* = < 0.0001). **B** Epigenetic Library (TargetMol) on doxycycline induced RIVA cells. Hits are compounds that significantly inhibited cell viability and compounds that had an increase or decrease in viability two standard deviations from the mean of the difference between BCL10 S136X and vector after 72 h of 10 μM drug treatment. Targets in red highlight mutant-driven resistance, mutant-sensitive targets are in blue, and compounds that sensitized both vector and BCL10 S136X are in purple. **C** Schematic of hits from 4B. **D** Dose response viability assays of RIVA cells treated with selcidemstat (p=ns), tovorafenib (p = ns), ulixertinib (S136X: *p* = 0.0125, R58Q: *p* = 0.0103), JNKi (p = ns), duvelisib (S136X: *p* = 0.126, R58Q: *p* = 0.0264), idelalisib (S136X: p = ns, R58Q: *p* = 0.004), capivasertib (S136X: *p* = 0.0002, R58Q: *p* = 0.005), venetoclax (S136X: *p* = 0.008, R58Q: *p* = 0. 0.046), and AZD1208 (PIMi) (S136X: *p* = 0.019, R58Q: *p* = 0.040).

BN2/C1 cases have poor clinical responses to targeted inhibitors, including mutant BCL10-driven BTKi resistance [8, 12]. We confirmed both mutants drive resistance to covalent and noncovalent BTKis (Fig. 4A, Supplemental Fig. 4A), confirmed orthogonally in competition assays (Supplemental Fig. 4B). To more fully assess impact of BCL10 mutations on treatment resistance, we interrogated the 960-compound TargetMol Epigenetic Library. This drug screen was selected for containing a broad range of compounds targeting BCR-activated and other clinically relevant signaling pathways in DLBCL. We assessed RIVA with BCL10-S136X and empty vector and analyzed results by both differential and absolute effect of each compound on each genotype (Fig. 4B). Targets in red highlight mutant-driven resistance, mutant-sensitive targets are in blue, and compounds that sensitized both vector and BCL10-S136X are in purple (Fig. 4B, C, Supplemental Table 8). Because the library screen was performed at single drug concentrations, we employed serial dilution viability assessments with compounds against specific targets to validate results (Fig. 4D). A recent study showed *BCL6*, whose translocation is a BN2 hallmark, requires LSD1 for lymphomagenesis [26] and the LSD1 inhibitor seclidemstat is in cancer clinical trials (NCT04734990, NCT03600649). Vector and mutants were equally sensitive to seclidemstat in the sub-micromolar range. RAF inhibition was potentially active in BCL10 mutants in line with up-regulation of this pathway (Fig. 3D). Tovorafinib is a type II pan-RAF kinase inhibitor in clinical trials (NCT05566795) [27], but it showed no significant difference between the vector and mutant, while the ERK inhibitor, ulixertinib, was more active against the mutants (Fig. 4D, Supplemental Fig. 6). Overall, these compounds were poorly potent with EC_50_ > 10 µM.

The drug screen suggested mutant-mediated resistance to a variety of additional drug classes, including those with negative DLBCL clinical trial results, including PI3K/AKTis (idelalisib, duvelisib, capivasertib) and the BCL2i venetoclax, all of which are active against other B-lymphomas (Fig. 4D) [28, 29]. Inhibitors of mTOR were also not effective in this system (Supplemental Fig. 4C). We confirmed BCL10-mutant resistance also for PIM kinase. Immunoblotting showed upregulation of PIM2 and phosphorylated AKT which may explain some results (Supplemental Fig. 4D). These analyses therefore suggest activation of the CBM complex by BCL10 mutations play key roles in clinical resistance to specific drug classes.

### BTK inhibition sensitizes BCL10-mutant tumors to venetoclax in vitro and in vivo

**Fig. 5:**
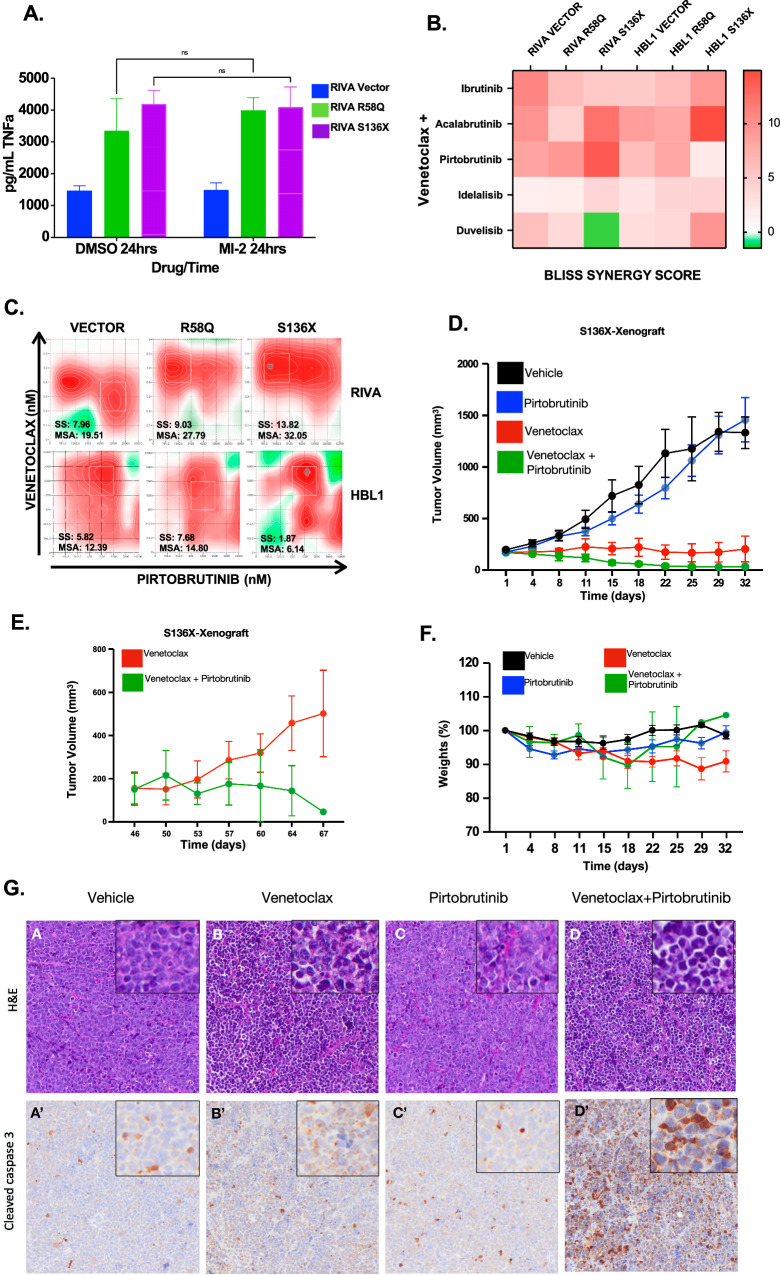
The combination of venetoclax and pirtobrutinib synergistically kills BCL10 mutant cells in vitro and in vivo. **A** ELISA assay of TNFα for cell supernatants in RIVA treated compared to untreated with MI-2 (250 nm × 24 h). **B** Heatmap of Bliss Synergy Scores of RIVA and HBL1 cells treated with venetoclax combined with either Bruton’s Tyrosine Kinase inhibitors or PI3 Kinase inhibitors. **C** Synergy assessments of venetoclax plus pirtobrutinib in doxycycline induced RIVA and HBL1 cells with reported synergy score (SS) and most synergistic area (MSA) values. **D** Tumor volumes of cell line xenograft of RIVA S136X cells in NSG mice treated with single-agent (Venetoclax; 10 mg/kg or Pirtobrutinib;30 mg/kg) and combination therapy. **E** Tumor volumes of venetoclax and combination therapy after completion of treatments. **F** Mouse weights per treatment group across experimental groups. **G** Hematoxylin and Eosin (H&E) (**A**–**D**) staining (upper panel) and immunohistochemical staining cleaved caspase 3 (**A**’–**D**’) (lower panel) on tumors derived from animals treated with either single agent venetoclax/ pirtobrutinib or the combination.

Direct inhibition of MALT1 is an emerging strategy to overcome BCR signaling [8, 30]. MALT1 protease inhibitors include the tool compound MI-2 and the clinical compound safimaltib (NCT03900598). ABC-DLBCL cells with BCL10 mutants upregulated MALT1 activity as demonstrated by increased cleavage of substrates A20 and CYLD, and these cells retained sensitivity to both MI-2 and safimaltib (Supplemental Fig. 5A, B). However, feedback activation of PI3K-AKT signaling, as previously described [31], and phosphorylation of ERK1/2 were consistent potentially confounding effects of these drugs (Supplemental Fig. 5C). Moreover, MALT1 inhibitors had no impact on TNFα production (Fig. 5A). These observations likely reflect MALT1’s protease-independent scaffolding functions promoting downstream signaling [32], demonstrating a need for novel strategies. We tested various clinical compounds up and downstream of CBM in combination with MALT1i. These suggested promise for BTKis and PI3Kis (Supplemental Fig. 5D). However, though MALT1i re-sensitized BCL10 mutants to inhibitors that the mutants initially showed resistance to, like BTKis and PI3Kis, the combinations were primarily additive. MALT1i-BTKi combinations showed promise in mantle cell lymphoma [33], but we were underwhelmed by the combination in BCL10-mutant DLBCL systems. Seclidemstat in combination with BTKis or MALT1is showed no evidence of synergy (Supplemental Fig. 5E).

We therefore sought alternatives with focus on overcoming protection from apoptosis. Combinations of venetoclax and BTKis, specifically ibrutinib, have been tested in CLL, where durable efficacy may occur [34, 35]. Given that BCL10 mutants upregulate BCL2 family proteins (Fig. 1C, Supplemental Fig. 4E, F) and that apoptosis factors were strongly upregulated in GSEA (Fig. 1D, Supplemental Fig. 4G), we tested venetoclax with BTKis and PI3Kis. Strikingly, despite resistance to all three drug classes as single agents (Fig. 4A, D), the combination of venetoclax with BTKis or PI3Kis was additive or synergistic regardless of mutation status (Fig. 5B, C). Synergy (Bliss Synergy Score > 10) was notable in BCL10-S136X where the combinations of venetoclax with acalabrutinib or pirtobrutinib were synergistic (Fig. 5B, C, Supplemental Fig. 7A, Supplemental Table S9). Synergy scores were lower for the PI3K inhibitors idelalisib and duvelisib (Fig. 4B). In addition, these drugs with regulatory approval history in indolent B-cell lymphomas either came off the market (idelalisib) or are under black-box warning for treatment-related mortality (duvelisib) associated with immune-mediated toxicities, and we did not pursue them further. We tested also the venetoclax combination with AKTi capavistertib, which is under clinical evaluation in B-lymphomas (NCT05008055), revealing moderate additivity (Supplemental Fig. 7B). Inhibitors of mTOR alone or combined with other therapies did not achieve significant success in DLBCL clinical trials despite strong preclinical promise but were not tested in specific mutant contexts [36–38]. Increased availability of these drugs in low-resource settings prompted us to test the combination of rapamycin plus venetoclax against BCL10-mutant-driven systems, but we saw no strong increase in induction of apoptosis (Supplemental Fig. 7C). Based on these factors, we focused on the BTKi/BCL2i combination moving forward.

Though ibrutinib is the most used BTKi to date, pirtobrutinib is a newer non-covalent, highly selective BTKi recently approved for r/r mantle cell lymphoma and CLL with reduced toxicity [39–41]. Venetoclax plus pirtobrutinib is active in CLL [42, 43] but has not been explored in DLBCL and we wanted to assess efficacy in vivo. RIVA cells with inducible BCL10 S136X were engrafted in NSG mice, and animals were fed food containing doxycycline which activated expression (Supplemental Fig. 9). Baseline RIVA cells are highly sensitive to venetoclax (Fig. 4D) [44], and even with the S136X mutant constitutively expressed, these tumors showed impaired growth in response to the single agent (Fig. 5D). Venetoclax (10 mg/kg) plus pirtobrutinib (30 mg/kg) by contrast caused complete regression of tumors rather than stability, and treatments were stopped at D32. Subsequent followup showed re-induction of tumor growth in the venetoclax single-agent group after D50, while this never occurred for ven+pirto (Fig. 5E). There were no differences in toxicities indicated by mouse average weights (Fig. 5F). End-treatment pathologic assessment revealed induction of cleaved caspase-3 in combination-treated tumors (Fig. 5G). We therefore find BTK retains key roles promoting drug resistance even in the context of direct CBM activation by BCL10 mutations.

### Key venetoclax resistance factors retain dependence on BTK for expression

**Fig. 6:**
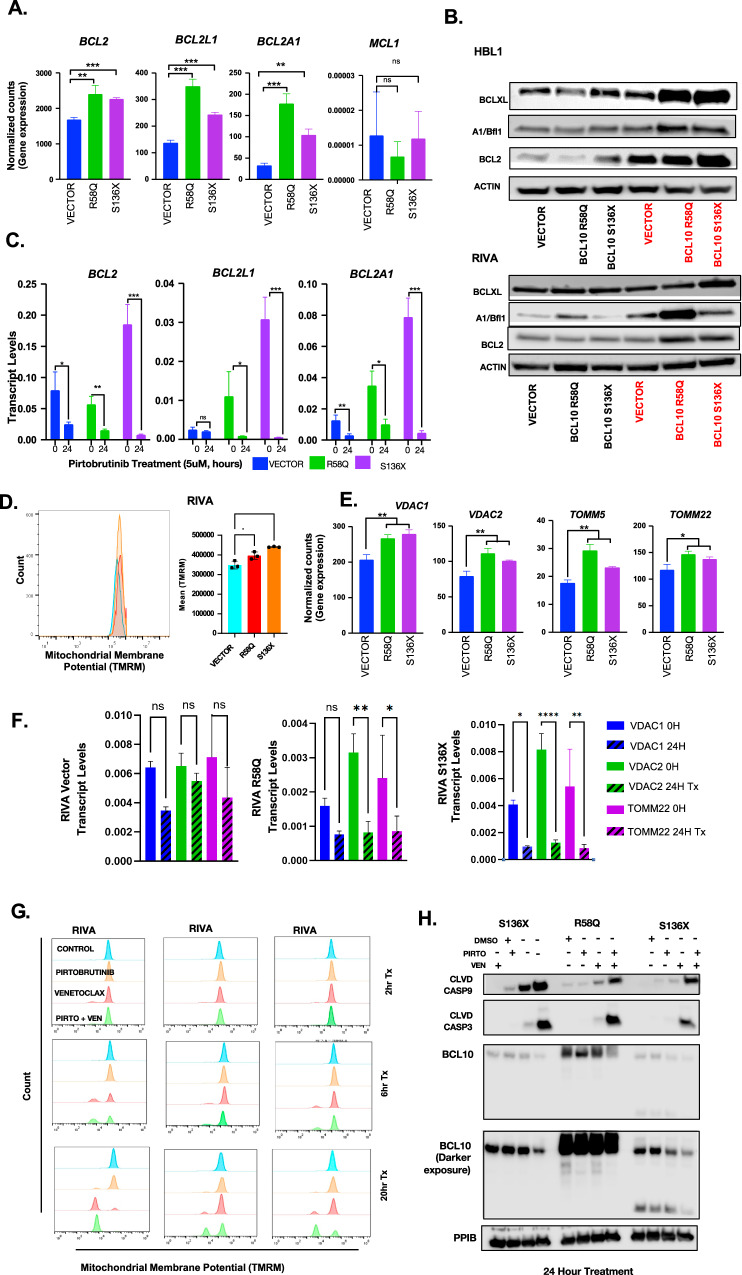
BCL10 mutants upregulate key resistance pathways. **A** Normalized counts from gene expression data for BCL2 family genes *BCL2, BCL2L1*, and *BCL2A1* in the BCL10 mutants. **B** Western blot of proteins implicated in venetoclax resistance including BCL-xL, BFL1, and BCL2 in doxycycline induced RIVA cells. **C** Transcript levels of doxycycline induced RIVA cells treated with DMSO or 5uM pirtobrutinib for 24 h: BCL2 (vector: *p* = 0.0325, R58Q: *p* = 0.005, S136X: *p* = 0.0006), BCL2L1 (vector: p=ns, R58Q: *p* = 0.0484, S136X: *p* = 0.0007), BCL2A1 (vector: *p* = 0.0087, R58Q: *p* = 0.0121, S136X: *p* = 0.0005). **D** Mitochondrial membrane potential flow cytometry assay in RIVA vector compared to RIVA R58Q and S136X at baseline. **E** Time course for BCL-xL, BFL1, and BCL2 in 5 μM pirtobrutinib treated RIVA cells. **F** Transcript levels of VDACs and TOMM22 after 24-h treatment with pirtobrutinib in RIVA cells. **G** Mitochondrial membrane potential flow cytometry assay in RIVA vector compared to RIVA R58Q and S136X after treatment with DMSO, pirtobrutinib, venetoclax or the combination for 2, 6 or 20 h. **H** Western blot of doxycycline induced RIVA cells treated with DMSO, venetoclax, pirtobrutinib or the combination for 24 h.

Increased expression of BCL2 family members including MCL1, BCL2L1 (BCL-xL), BCL2A1 (BFL1), and BCL2 itself drives resistance to single-agent venetoclax in various cancers [10, 44–48]. We assessed our RNA-seq data and found BCL10-mutant samples significantly increase expression of *BCL2L1* (S136X *p* = 0.0001, R58Q *P* = 0.0002), *BCL2A1*(S136X *p* = 0.0011, R58Q *p* = 0.0005), and *BCL2* (S136X *p* = 0.0001, R58Q *p* = 0.0074), while *MCL1* was unchanged (Fig. 6A), confirmed at the protein level (Fig. 6B). We next treated RIVA *BCL10* systems with 5 µM pirtobrutinib, which is EC_50_ for the vector control cells (Fig. 4A). Strikingly, even though this concentration is well below EC_50_ for BCL10-mutant lines, all showed dramatic decline in expression of these factors (Fig. 6C). Resistance to pirtobrutinib by the BCL10 mutant lines despite the sharp declines in these protective factors suggests they exist at a higher threshold for apoptosis due to constitutive CBM activation. Assessment of mitochondrial outer membrane potential which is a measure of oxidative phosphorylation (OXPHOS) activity by cytometry confirmed significantly higher potential in both S136X and R58Q cells (Fig. 6D), associated with increased VDACs (mitochondrial inner membrane voltage-dependent anion channels) and TOMM (outer membrane) proteins that contributes mitochondrial membrane potential through OXPHOS activity (Fig. 6E) [49]. A higher apoptotic threshold could promote resistance to multiple drug classes seen in Fig. 4, and we hypothesized pirto+ven is a one-two punch that overcomes this to explain synergy. To test this, we examined mitochondrial membrane potential across time points of treatment with pirtobrutinib, venetoclax, or both, demonstrating delayed, compared to control, but nonetheless strong mitochondrial depolarization in the BCL10-mutant systems in response to only the combination (Fig. 6F, Supplemental Fig. 10A). Pirtobrutinib alone was effective in reducing VDAC levels, likely sensitizing the cells to venetoclax (Supplemental Fig. 10B). Western blotting confirmed induction of apoptotic markers that in BCL10-mutant systems was true only in response to the combination (Fig. 6G, Supplemental Fig. 8). Therefore, even though CBM activation by BCL10 mutants promotes BTKi resistance, the enzyme nonetheless maintains key roles protecting tumor cells from apoptosis, overcome by inhibition and informing a highly promising combination with venetoclax.

### Multiple downstream pathways retain BTK dependence in BCL10-mutatnt tumors

**Fig. 7:**
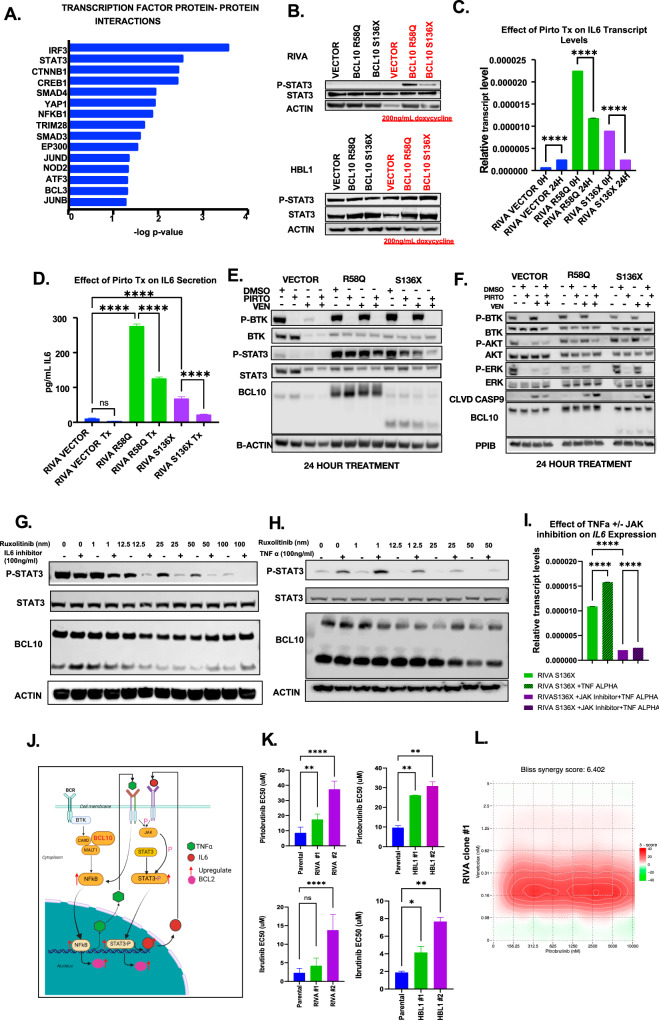
The mechanism of the combination of pirtobrutinib and venetoclax. **A** Upregulated transcription factor protein-protein interactions in genes upregulated by the BCL10 mutants probed through the Enrichr website (*p* < 0.05). **B** Western blot showing phosphorylated STAT3 and total STAT3 in HBL1 and RIVA cells containing BCL10 mutants. **C** IL6 transcript levels of RIVA vector and mutants treated with pirtobrutinib for 24 h (*p* < 0.0001). **D** IL6 cytokine levels in RIVA vector and mutants supernatant treated/untreated with 10uM pirtobrutinib for 24 h. **E**, **F** Western blot of doxycycline induced RIVA cells treated with DMSO, venetoclax, pirtobrutinib or the combination for 24 h. **G** Western Blot analysis of RIVA S136X cells treated with increasing concentrations of ruxolitinib with or without tocilizumab (IL6 inhibitor). **H** Western Blot analysis of RIVA S136X cells treated with increasing concentrations of ruxolitinib with or without the addition of TNFa. **I** IL6 transcript levels of RIVA S136X cells treated with TNFa alone or with ruxolitinib. **J** Schematic of TNFa**/**IL6/JAK/STAT signaling in BCL10 mutant cells. **K** Dose response viability assays of RIVA and HBL1 cells treated with pirtobrutinib (HBL1 clone1: *p* = 0.0027, HBL1 clone2: *p* = 0.0013, RIVA clone1: *p* = 0.0029, RIVA clone2: *p* < 0001) and ibrutinib (HBL1 clone1: *p* = 0.0401, HBL1 clone2: *p* = 0.0028, RIVA clone1: *p* = 0.3089, RIVA clone2: *p* < 0001), clones1 and 2 are the mutation (S136X) engineered at BCL10 gene locus. **L** Synergy assessments of venetoclax plus pirtobrutinib in endogenous BCL10 mutants (S136X) with reported synergy score.

To elaborate these findings, we examined additional signaling downstream of BTK. Top pathways deregulated by BCL10 mutants included IL6-JAK-STAT3 (Fig. 1D), and the R58Q mutant in particular showed increased IL6 production (Fig. 2C, E). STAT3 like NF-kB upregulates anti-apoptotic genes [50, 51]. Analysis of protein-protein interactions in Enrichr demonstrated that among genes upregulated by BCL10 mutants, STAT3 had the second highest level of interaction highlighting a strong relationship in the BCL10-mutant dataset (Fig. 7A). Interactions with JUN family proteins (JUND, JUNB) were also increased, along with NFKB1. Since potently oncogenic STAT3 activation is downstream of IL6 engagement with its receptor, we explored whether STAT3 increased activation in BCL10-mutant systems. Both S136X and R58Q mutants demonstrate significantly increased baseline STAT3-S701 phosphorylation in both RIVA and HBL1 (Fig. 7B). IL6 expression is activated by NF-kB transcription factors and therefore could be independent of BTK in BCL10-mutant systems, but multiple other signaling mechanisms also regulate its production and secretion [52]. We therefore tested the effect of pirtobrutinib and found strikingly that *IL6* expression (Fig. 7C) and production of the mature cytokine (Fig. 7D), like BCL2 family members in Fig. 6, retain dependence on BTK in both BCL10-mutant contexts. STAT3 activation similarly retains sensitivity to pirtobrutinib (Fig. 7E). Because many other signals may impact IL6 production, STAT3 activation, and expression of anti-apoptotic factors, we looked also at PI3K/AKT and MAPK, both of which may mediate venetoclax resistance [53]. Activating phosphorylation of both AKT and ERK1/2 showed pirtobrutinib sensitivity across BCL10 mutants (Fig. 7F). The combination of venetoclax and ibrutinib was sensitive in all RIVA cells, particularly in the S136X mutant and showed similar but less durable effects as the suppression of phosphorylated ERK and phosphorylated AKT was not sustained at 24 h (Supplemental Figs. 11, 12). Apart from the PI3K/AKT and MAPK pathways, we also investigated the role of TNFα-mediated upregulation of IL6. Inhibition of IL6 (Tocilizumab) or the addition of TNFα along with the JAK inhibition (Ruxolitinib) resulted in the downregulation and upregulation, respectively, of STAT3 phosphorylation, directly explaining the observed upregulated IL6 levels in the mutants (Fig.7G–J). Our findings suggest that upregulated cytokine-mediated signaling downstream of BCL10 mutants contributes to drug resistance in part through enhanced STAT3 activation and pirtobrutinib treatment effectively inhibited this pathway, thereby sensitizing cells to venetoclax. Finally, we wanted to establish through CRISPR/Cas9 editing, systems with monoallelic mutation of endogenous *BCL10*, as in patient tumors, to cross-compare with tet-on expression. As previously reported [8], these systems were challenging to establish with consistent behavior, but we eventually isolated single-cell clones of RIVA and HBL1 with S136X expression from successful editing of single endogenous *BCL10* alleles (Supplemental Fig. 13). These replicated key findings of BTKi resistance overcome by the combination with venetoclax (Fig. 7K, L). In sum, despite BTK-independent activation of NF-kB and promotion of BTK inhibitor resistance, BCL10-mutant tumors retain dependence on the enzyme for sustained activation of key pathways.

## Discussion

Biomarker-informed use of specific signaling inhibitors underlies clinical paradigms in blood cancers like acute myeloid leukemia and multiple solid tumors [54–57]. DLBCL has defied such precision-medicine approaches. Partly, this is for a positive reason—efficacy of standard combination chemoimmunotherapy against newly diagnosed DLBCL even at advanced stages. R-CHOP (rituximab, cyclophosphamide, doxorubicin, vincristine, prednisone) and similar is generally used regardless of frontline risk profile and cures ~60% [58, 59]. Even rel/ref patients often have curative-intent subsequent-line options, including salvage chemotherapy with stem-cell transplant consolidation and CD19-directed chimeric-antigen receptor (CAR) T-cells [7, 60, 61]. Clinical decision making among these options remains largely uninformed by specific biomarkers, and unfortunately, most rel/ref patients ultimately will be failed by them. Precision-medicine approaches to improve outcomes face several barriers. These include clinical factors like potential for rapid progression and/or compromised patient performance status related to prior treatments. Disease heterogeneity, however, is generally seen as the greatest challenge [62, 63]. At least 5–7 molecular subgroups exist at a level of recurrent patterns of single-gene alterations [9–11], implicating disparate mechanisms that are frequently undruggable, like *MYC* rearrangements or constitutively activated NF-kB transcription factors. Addition of ibrutinib to frontline therapy in the randomized PHOENIX trial was a precision-medicine attempt to reduce frequency of rel/ref disease among traditionally high-risk ABC COO patients but was unsuccessful except in post-hoc analysis of younger patients in two molecular subgroups [12, 64]. Patients in the BN2 group, the subject of this study, specifically did not benefit.

To better understand these discouraging observations, we modeled drug resistance driven by *BCL10* mutations that cluster in BN2/Cluster 1. Results validate and elaborate on BTK-inhibitor resistance driven by these lesions [8]. We find additional classes of drug that notably have failed in DLBCL clinical trials are thwarted by these alterations. Cellular models supported by gene-expression data from clinical cases demonstrate a cytokine-reinforced positive feedback loop driving lymphomagenesis in the mutant-BCL10 context. Our study was not designed to elucidate differences between the CARD-domain R58Q mutation and C-terminal truncations, focusing instead on unifying mechanisms of lymphomagenesis and strategies to overcome them. We observed diffuse activation of transcription factors, some directly downstream from CBM like NF-kB and JNK molecules, but most of which appear more related to secondary engagement of cytokine-mediated signaling, including JAK/STAT, PI3K/AKT/mTOR and MAPK. We validated previous findings that BCL10 mutant-driven tumors retain sensitivity to disruption of MALT1 paracaspase activity and found also potential promise to inhibit the epigenetic eraser LSD1, both of which are targetable with clinical compounds. Further assessment, however, left us underwhelmed by these approaches both alone and in various combinations. We focused instead on combined targeting of BTK and BCL2, even though BCL10 mutants drove resistance to both individually. Venetoclax, like ibrutinib, long failed to find role in DLBCL management [22, 65, 66], and we reveal direct CBM complex activation as a likely explanation in relevant cases. BCL10-mutant tumors showed strong up-regulation of not only BCL2 family members, BCL2L1 (BCL-xL) and BCL2A1 (BFL1) but also OXPHOS pathway that drive venetoclax resistance. Strikingly, however, these effects retained sensitivity to BTK inhibition. The non-covalent BTK inhibitor pirtobrutinib, in particular, markedly decreased BCL2L1, BCL2A1, and BCL2 itself across BCL10 WT and mutant contexts. Pirtobrutinib treatment also affects the transcript levels of VDACs contributing high OXPHOS activity. BTK therefore retains key roles protecting cells from apoptosis even when dispensable for CBM activation (Graphical Abstract).

As our laboratory investigations were underway, so too was clinical evaluation of the novel combination regimen ViPOR [5]. Designed as low-toxicity salvage therapy combining oral agents and the second-generation anti-CD20 monoclonal antibody obinutuzumab, ViPOR as in our results combines venetoclax and a BTK inhibitor (ibrutinib) along with lenalidomide and prednisone. ViPOR becomes the first DLBCL drug regimen able to promote significant rates of complete response while excluding traditional chemotherapy agents completely. Suggestively, its activity was significantly higher in non-GCB cases compared to GCB. We believe these results may provide clinical validation of our mechanistic studies, even though they cannot yet be linked to the highly specific biomarker of BCL10 gain-of-function, pending more widespread use and correlative studies.

Limitations of this report include a lack of studies that capture effects on lymphoma microenvironments. We studied only autocrine effects of the diffuse activation of cytokine production we observed. Immunocompetent models are necessary to interrogate the undoubtedly significant paracrine effects these cytokines will have on neighboring non-malignant cells. We have generated a cre-inducible *BCL10*-S136X genetically engineered mouse that is the focus of work in progress. Results from these studies will follow as a later report.

Ultimately, we believe the combination of pirtobrutinib plus venetoclax deserves evaluation in DLBCL patients to assess clinical utility of our observations. Dramatic activity of BTK inhibitors in some indolent B-NHLs, CLL in particular, reflect an overall simpler oncogenic milieu in these diseases compared to DLBCL. The phase 3 BRUIN CLL-322 trial is currently evaluating the combination of pirto+ven and rituximab in untreated CLL, providing helpful safety and side effect data for the combination [67]. Pirto+ven is a promising strategy to overcome the greater complexity and should also be examined in the context of immunotherapy approaches like existing and next-get CAR-T cells and immune effector-engaging multivalent antibodies.

## Supplementary information

Table4, Table5, Table6, Table7, Table8

Supplementary Appendix

## Data Availability

Data were generated by the authors or were analyzed from primary sources cited and are available as needed upon request from the corresponding author. RNA sequencing data we generated are available from Gene Expression Omnibus (https://www.ncbi.nlm.nih.gov/geo/) under GSE267421.
