# Supplementary material for: Bruton’s tyrosine kinase inhibition re-sensitizes multidrug-resistant DLBCL tumors driven by BCL10 gain-of-function mutants to venetoclax
Source: Blood Cancer J. 2025 Feb 2;15(1):9. doi: 10.1038/s41408-025-01214-y (PMC11788437; doi:10.1038/s41408-025-01214-y)
Supplement: Supplementary file 2 — Supplementary Appendix [file 41408_2025_1214_MOESM2_ESM.docx]

**Supplementary Appendix**

**Table of Contents**

***SUPPLEMENTARY TABLES***

**Table S1.** Compounds used in cell-based experiments.

**Table S2.** Antibody List

**Table S3.** Primers List

**Table S4.** Jensen Compartment analysis – significant complexes (in Supp. Tables spreadsheet)

**Table S5.** GSEA results – significantly enriched gene sets (in Supp. Tables spreadsheet)

**Table S6.** GO analysis – significantly deregulated ontologies (in Supp. Tables spreadsheet)

**Table S7.** ENCODE transcription factor analysis results (in Supp. Tables spreadsheet)

**Table S8.** TargetMol library screen results (in Supp. Tables spreadsheet)

**Table S9.** Synergy score for the drug treatments.

***SUPPLEMENTARY FIGURES***

**Figure S1.** Inducible BCL10 mutations affect NF-kB activity and CBM complex components.

**Figure S2.** Differentially expressed genes in BCL10 mutants compared to vector.

**Figure S3.** Human cytokine array

**Figure S4.** BCL10 mutants are resistant to BTKis.

**Figure S5.** Inhibition of MALT1 sensitizes BCL10 mutants but does not overcome all oncogenic signaling.

**Figure S6**. Single agent venetoclax treatment in DLBCL cell lines.

**Figure S7**. Synergy of covalent BTKis plus venetoclax in RIVA cells and apoptosis assay for the combination of rapamycin and venetoclax.

**Figure S8**. Sensitivity to Pirtobrutinib and Venetoclax combination is retained and enhanced in BCL10 mutants.

**Figure S9.** RIVA S136X tumor xenograft. Western blot of cell controls with the xenograft tumor.

**Figure S10.** Mitochondrial membrane potential after treatment.

**Figure S11.** Competition Assay of Ibrutinib and Venetoclax in RIVA cells.

**Figure S12.** Combination therapy of ibrutinib and venetoclax in BCL10 mutant RIVA cells.

**Figure S13.** CRISPR/Cas9 editing at endogenous BCL10 locus.

**Supplementary Tables 1-3, 9 (all others in separate spreadsheet)**

**Table S1.** Compounds/drugs in cell-based experiments.

| **S.no** | **Compound/drugs name** | **Catalog no/Source** |
| --- | --- | --- |
| **1** | MI-2 | S7429/ SelleckChem |
| **2** | AZD1208 | 7104/ SelleckChem |
| **3** | Ibrutinib | HY-10997/ MedChemExpress |
| **4** | Acalabrutinib | HY-17600/ MedChemExpress |
| **5** | Pirtobrutinib | HY-131328/ MedChemExpress |
| **6** | Duvelisib | HY-17044/ MedChemExpress |
| **7** | Idelalisib | HY-13026/ MedChemExpress |
| **8** | Capivasertib | HY-1543/ MedChemExpress |
| **9** | Venetoclax | HY-15531/ MedChemExpress |
| **10** | Safimaltib | HY-139399/ MedChemExpress |
| **11** | Tovorafenib | HY-15246/ MedChemExpress |
| **12** | JNK-IN-8 | HY-13319/ MedChemExpress |
| **13** | Seclidemstat | HY-103713/ MedChemExpress |
| **14** | Ulixertinib | HY-15816/ MedChemExpress |
| **15** | Ruxolitinib | HY-50856/ MedChemExpress |
| **16** | TNFalpha | Thermofisher |
| **17** | Tocilizumab | HY-P9917/MedChemExpress |

**Table S2. Antibody List.**

| **S.No.** | **Antibody** | **Dilution** | **Application** | **Source/catalog no.** |
| --- | --- | --- | --- | --- |
| **1** | CARD11 | 1:500 | Western blot | Abcam/ab11547 |
| **2** | MALT1 | 1:2000 | Western blot | Abcam /33921 |
| **3** | BCL10 | 1:1000 | Western blot | CST/4237S |
| **4** | phospho-p65 | 1:500 | Western blot | CST /3033S |
| **5** | P65 | 1:1000 | Western blot | CST/8242 |
| **6** | phospho-IKBα | 1:500 | Western blot | CST /9246 |
| **7** | IKBα | 1:1000 | Western blot | CST /4814S |
| **8** | CYLD | 1:500 | Western blot | CST /4495S |
| **9** | A20 | 1:500 | Western blot | CST /5630S |
| **10** | phospho-STAT1 | 1:500 | Western blot | CST /8826S |
| **11** | STAT1 | 1:1000 | Western blot | CST /14995S |
| **12** | phospho-STAT2 | 1:500 | Western blot | CST /88410S |
| **13** | STAT2 | 1:1000 | Western blot | CST /72604 |
| **14** | BCL2 | 1:2000 | Western blot | CST /4223S |
| **15** | BCL-xL | 1:1000 | Western blot | CST /2764S |
| **16** | Cleaved Caspase-9 | 1:1000 | Western blot | CST /52873S |
| **17** | A1-Bfl-1 | 1:1000 | Western blot | CST /64310S |
| **18** | PIM2 | 1:1000 | Western blot | CST /4730S |
| **19** | phospho-ERK | 1:500 | Western blot | CST /9106S |
| **20** | ERK | 1:1000 | Western blot | CST/ 4695S |
| **21** | phospho-BTK | 1:500 | Western blot | CST /87457S |
| **22** | BTK | 1:1000 | Western blot | CST/ 3533S |
| **23** | α-tubulin | 1:2000 | Western blot | CST /2125S |
| **24** | JNK | 1:1000 | Western blot | CST /4672S |
| **25** | phospho-JNK | 1:500 | Western blot | CST /4668S |
| **26** | c-JUN | 1:1000 | Western blot | CST /9165S |
| **27** | phospho-c-JUN | 1:500 | Western blot | CST /9261S |
| **28** | AKT | 1:1000 | Western blot | CST /4685S |
| **29** | phospho-AKT | 1:500 | Western blot | CST /4060S |
| **30** | GAPDH | 1:2000 | Western blot | CST /5174T |
| **31** | CYPB | 1:10000 | Western blot | ThermoFisher/ PA1-027A |
| **32** | Rabbit anti-goat IgG HRP-linked | 1:2000 | Western blot | Thermo Fisher #31402 |
| **33** | Goat anti-rabbit IgG, HRP-linked | 1:2000 | Western blot | CST/7074S |
| **34** | Horse anti-mouse IgG, HRP-linked | 1:2000 | Western blot | CST /7076S |
| **35** | Cleaved caspase 3 | 1:200/ 1:1000 | IHC/ Western blot | CST/ 9664 |
| **36** | Phospho-Stat3 | 1:500 | Western blot | CST/9138 |
| **37** | Stat3 | 1:1000 | Western blot | CST/4904 |

**Table S3. Primer List (qPCR).**

| BCL-xL | Fw 5’ GGAGAACGGCGGCTGGGATA 3’ |
| --- | --- |
|  | Rv 5’ GGCCACAGTCATGCCCGTCA 3’ |
| BCL2 | Fw 5’ AGGCTGGGATGCCTTTGTGGAA 3’ |
|  | Rv 5’ CAAGCTCCCACCAGGGCCAAA 3’ |
| MCL1 | Fw 5’ AGGGCGACTTTTGGCCACCG 3’ |
|  | Rv 5’ TGCCTTGGAAGGCCGTCTCG 3’ |
| BCL2A1 | Fw 5’-TTACAGGCTGGCTCAGGACT-3’ |
|  | Rv 5’-AGCACTCTGGACGTTTTGCT-3’ |
| 18S | Fw 5’-TGACGGAAGGGCACCACCAG-3’ |
|  | Rv 5’-GCACCACCACCCACGGAATCG-3’ |

**Table S9. Bliss synergy score for the drug treatments.**

|  | RIVA VECTOR | RIVA R58Q | RIVA S136X | HBL1 VECTOR | HBL1 R58Q | HBL1 S136X |
| --- | --- | --- | --- | --- | --- | --- |
| Ibrutinib | 10.40 | 5.90 | 4.80 | 4.61 | 5.8 | 8.83 |
| Acalabrutinib | 9.27 | 4.14 | 12.10 | 8.39 | 7.64 | 14.88 |
| Pirtobrutinib | 7.96 | 9.03 | 13.82 | 5.82 | 7.68 | 1.87 |
| Idelalisib | 1.40 | 1.60 | 3.60 | 2.37 | 3.73 | 3.88 |
| Duvelisib | 5.68 | 3.33 | -1.47 | 3.03 | 3.39 | 9.19 |

**SUPPLEMENTARY FIGURES**

**
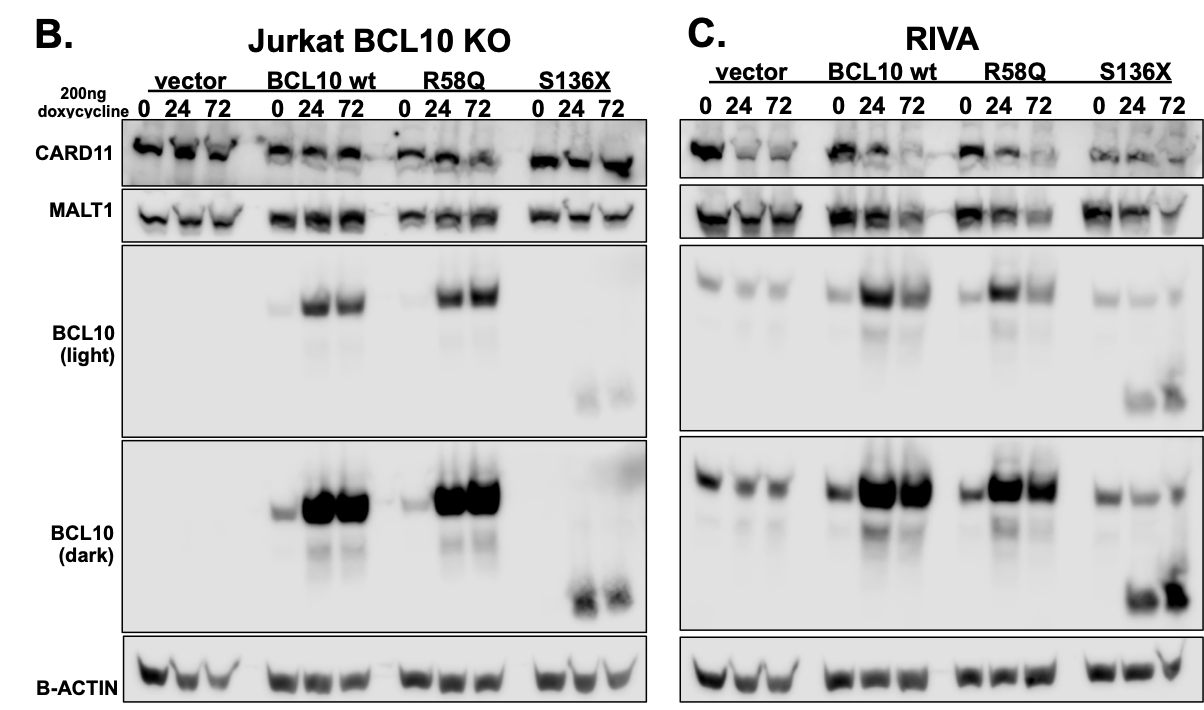

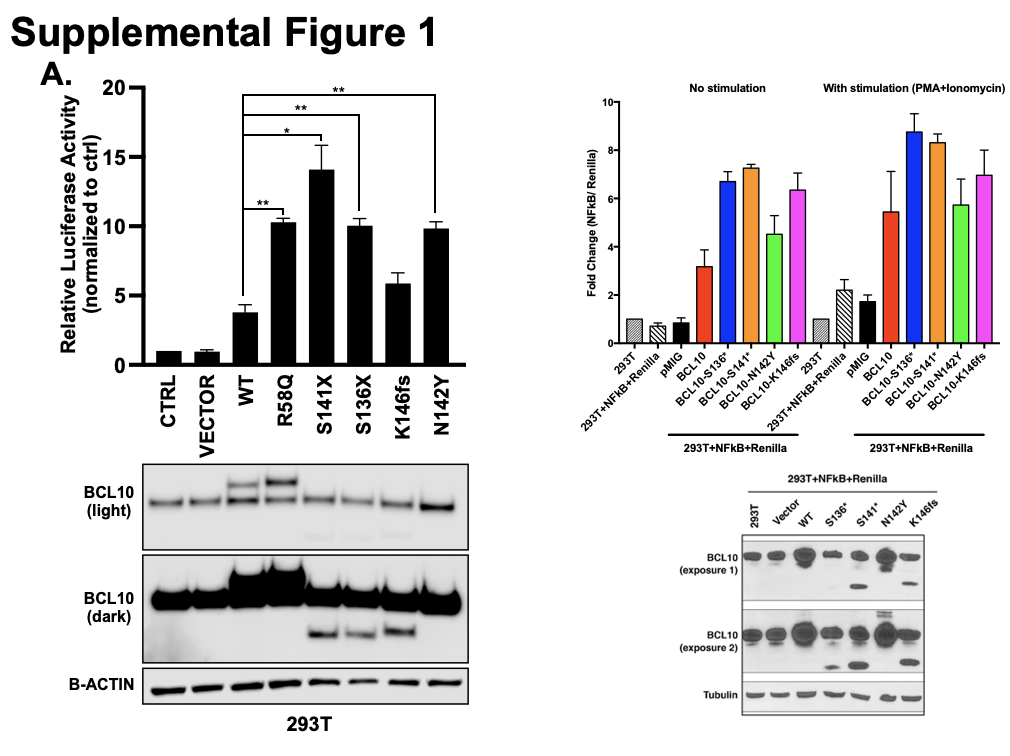
**

**
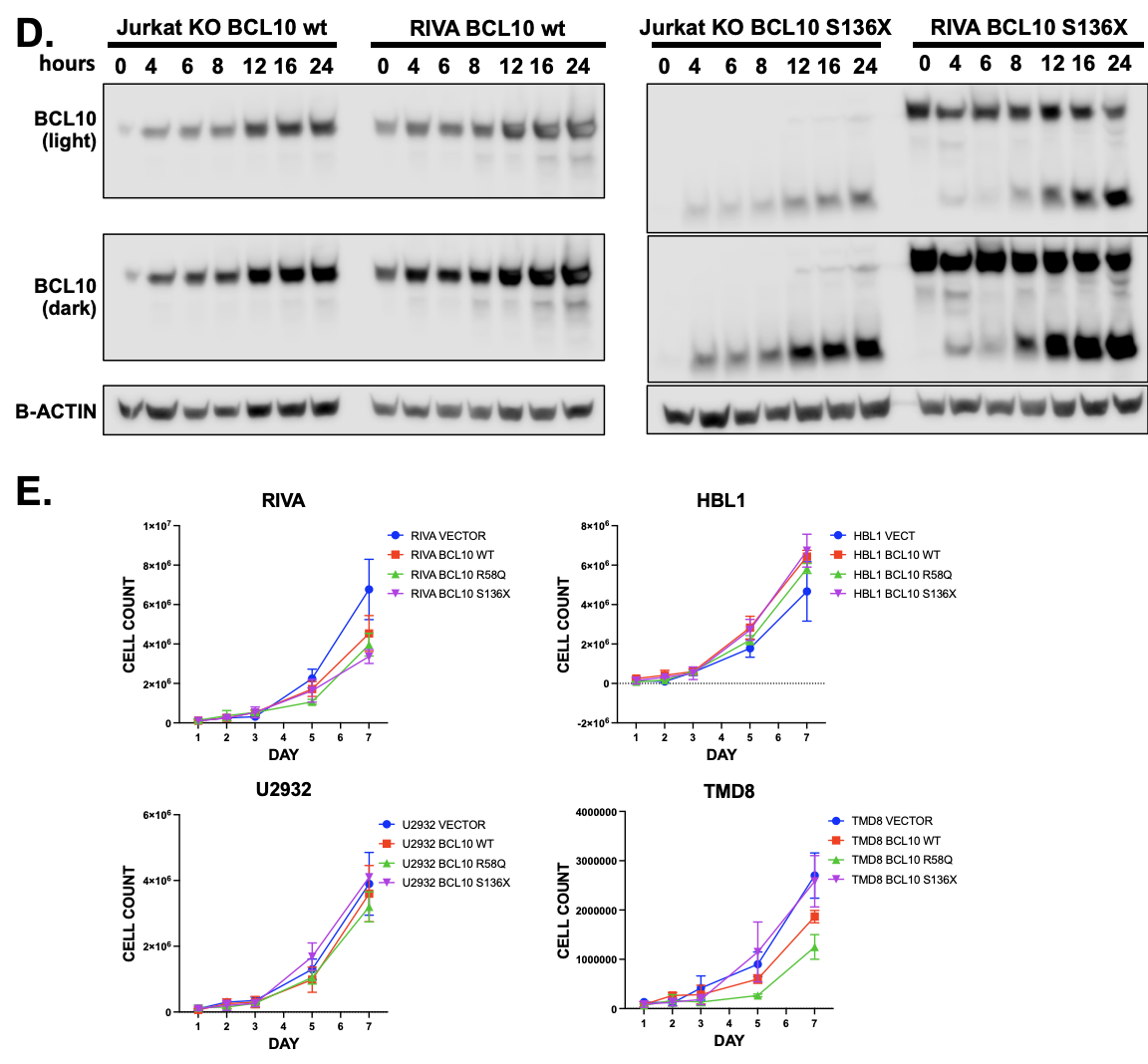
**

**
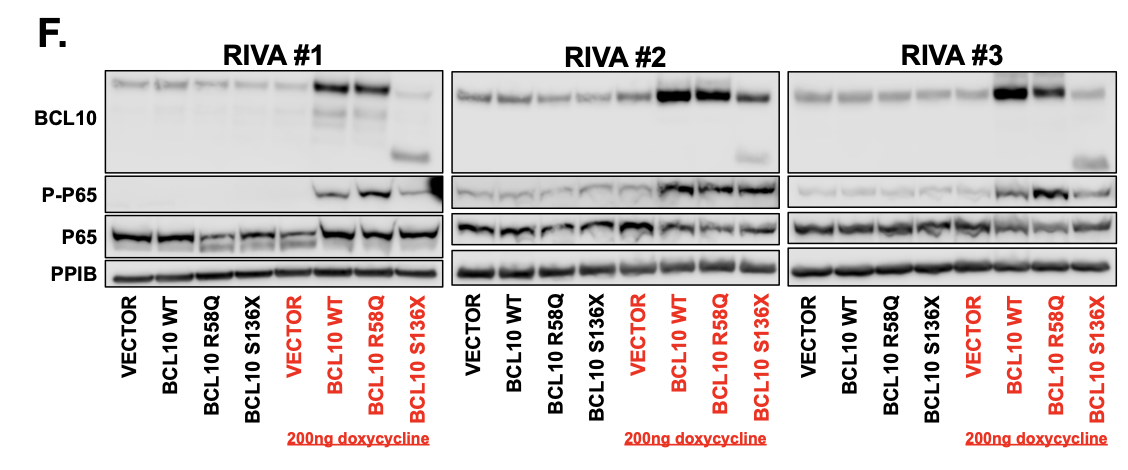
**

**Supplemental Figure 1 (two previous pages).** **Inducible BCL10 mutations affect NF-kB activity and CBM complex components.** **A.** Dual luciferase reporter assay in 293T cells with BCL10 wild type and mutant constructs. **B.** Dual luciferase reporter assay in 293T cells with BCL10 wild type and mutant constructs with and without stimulation by PMA and ionomycin. **C.** Western blot of Jurkat BCL10 KO cells and RIVA cells containing the BCL10 vectors induced with 200ng doxycycline for 0, 24, or 72 hours. **D.** Western blot of Jurkat BCL10 KO cells and RIVA cells containing the BCL10 WT or S136X induced with 200ng doxycycline for 0, 4, 6, 8, 12, 16 and 24 hours. **E.** Daily cell counts of doxycycline induced cells containing BCL10 vector, wildtype and mutants. **C.** EGFP-NFkB reporter assay in BCL10 mutant RIVA cells. **F.** Western blot of the triplicate samples of RIVA cells expressing BCL10 vector, wild type and mutants submitted for RNA sequencing.


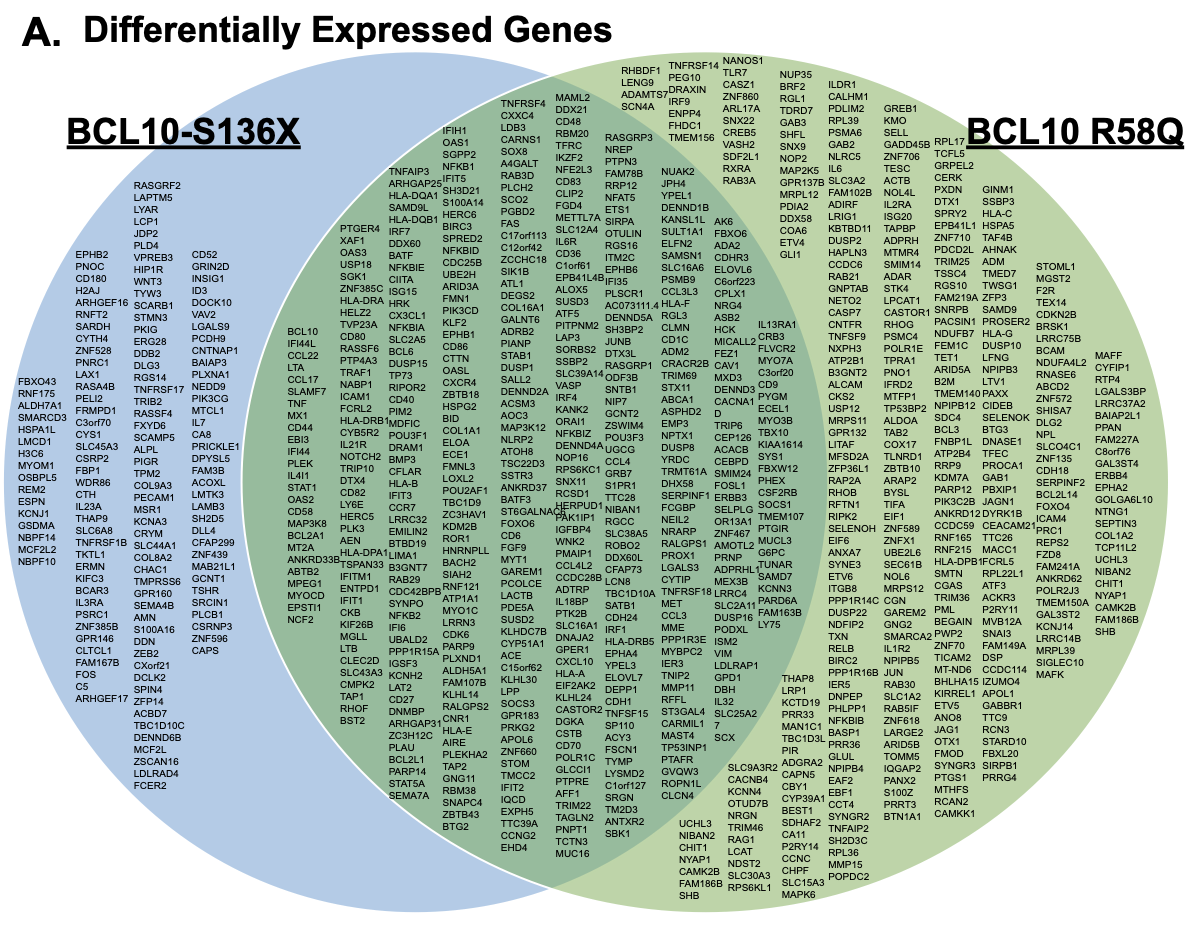


**Supplemental Figure 2. Differentially expressed genes in BCL10 mutants compared to vector. A.** Venn diagram of complete results of the differentially expressed genes (FC≤1.5).

**
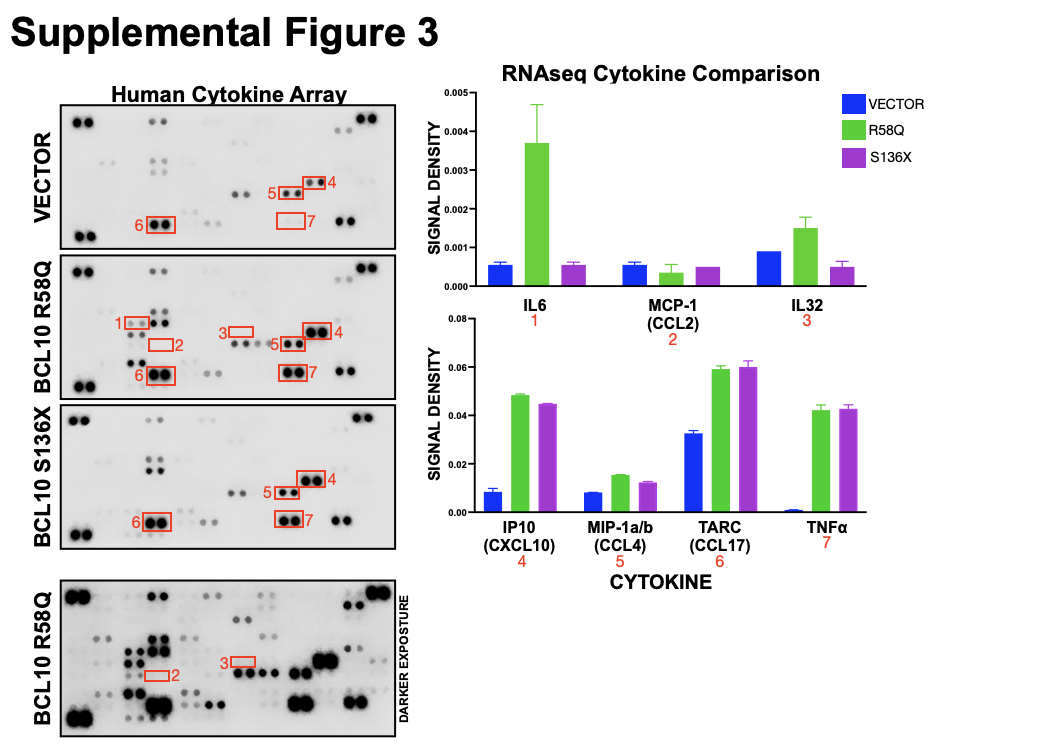
**

**Supplemental Figure 3. Human cytokine array.** Cell supernatant of RIVA cells treated with 200ng/ul of doxycycline for 72 hours and the Human XL Cytokine Array (R&D Systems: ARY022B) was performed according to manufacturer’s protocol. The results were analyzed using the LiCor system and signal density was graphed.

**Supplemental Figure 4 (next page). BCL10 mutants are resistant to BTKis. A.** Dose response viability assays of HBL1, U2932, and TMD8 cells treated with ibrutinib. **B.** HBL1 and RIVA cells containing a stably expressing GFP lentiviral vector containing empty vector, BCL10 wild type, R58Q and S136X were treated with ibrutinib over the course of 20 days. Cells were repeatedly treated, washed out and treated again with drug. GFP expression was reported. **C.** Dose response viability assays of HBL1 and RIVA cells treated with rapamycin. **D.** Western blot of RIVA and HBL1 cells induced with doxycycline for 24 hours and probed for phosphor-AKT, AKT, PIM2, and PPIB**. E.** qPCR of doxycycline-induced RIVA cells for BCL2 (S136X: p=0.0131, R58Q: p=ns), BCL2L1 (S136X: p=00010, R58Q: p=ns) and BCL2A1 (S136X: p=0.0008, R58Q: p=0.0177). **F.** Western blot of RIVA and HBL1 cells induced with doxycycline for 24 hours and probed for BCL2 and PPIB. **G.** GSEA analysis of the Hallmark Apoptosis pathway of the BCL10 mutants.

**
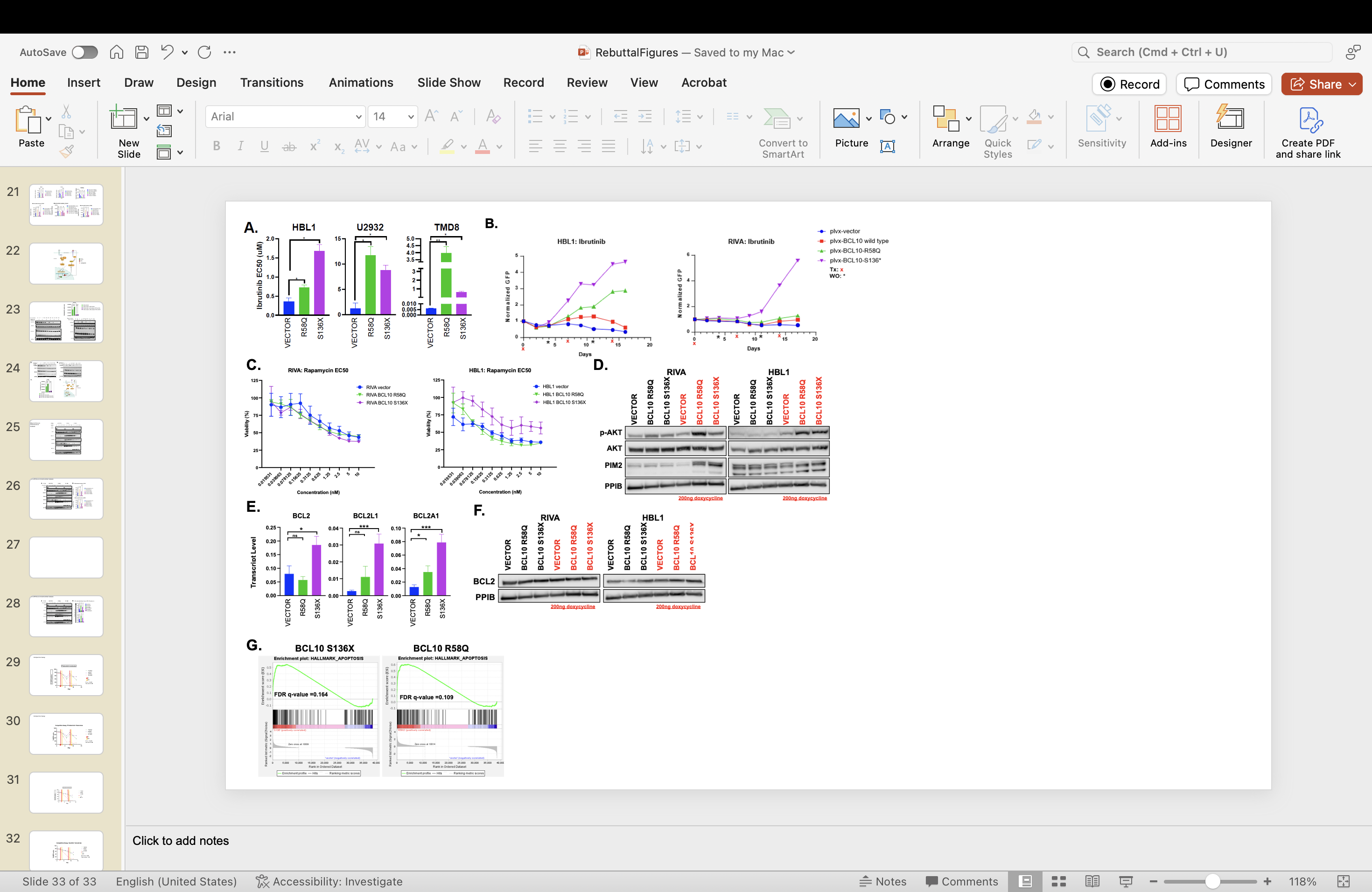
**

**
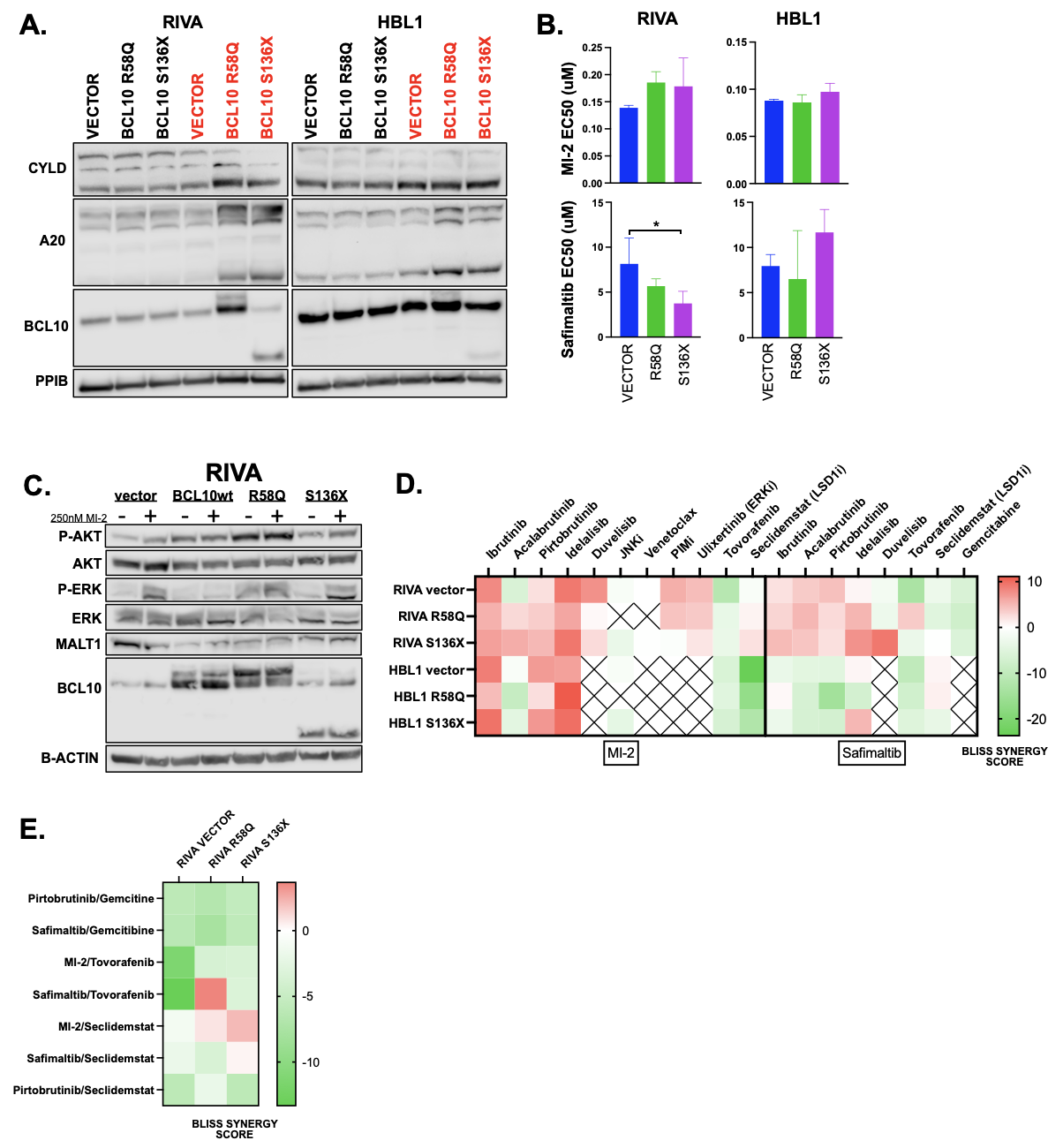
**

**Supplemental Figure 5. Inhibition of MALT1 sensitizes BCL10 mutants but does not overcome all oncogenic signaling. A.** Western blot of RIVA and HBL1 cells induced with doxycycline for 24 hours and probed for CYLD, A20, BCL10, and PPIB. **B** Dose response viability assays of RIVA and HBL1 cells treated with the MALT1 inhibitors MI-2 and safimaltib. **C.** Western blot of doxycycline induced RIVA cells treated with DMSO or MI-2 for 24 hours. **D. & E.** Heatmaps of Bliss synergy scores from doxycycline-induced RIVA cells treated with various drug combinations.


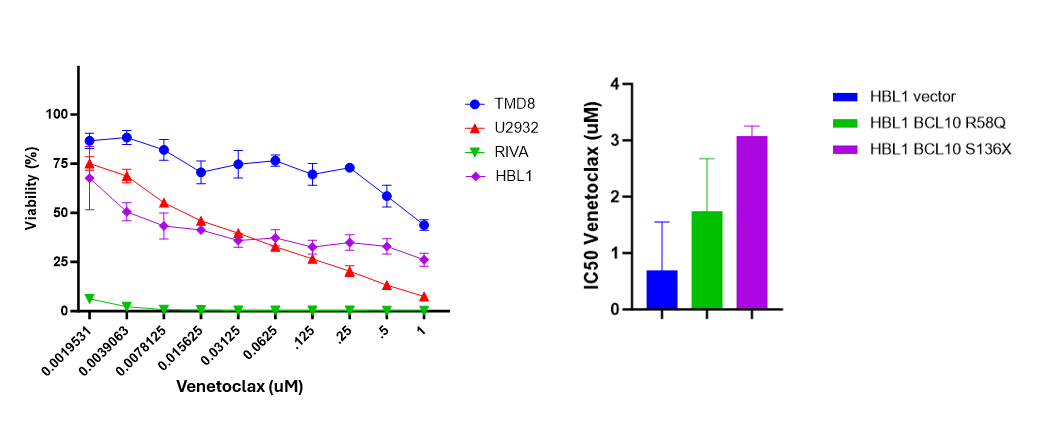


**Supplemental Figure 6.** Single agent venetoclax treatment in DLBCL cell lines and resistance in BCL10 mutants in HBL-1 cell lines.


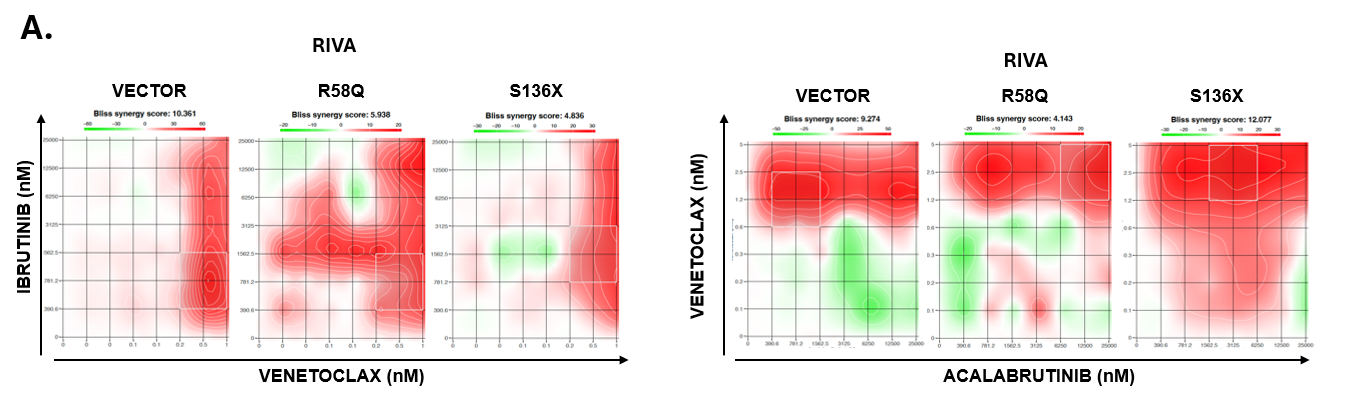


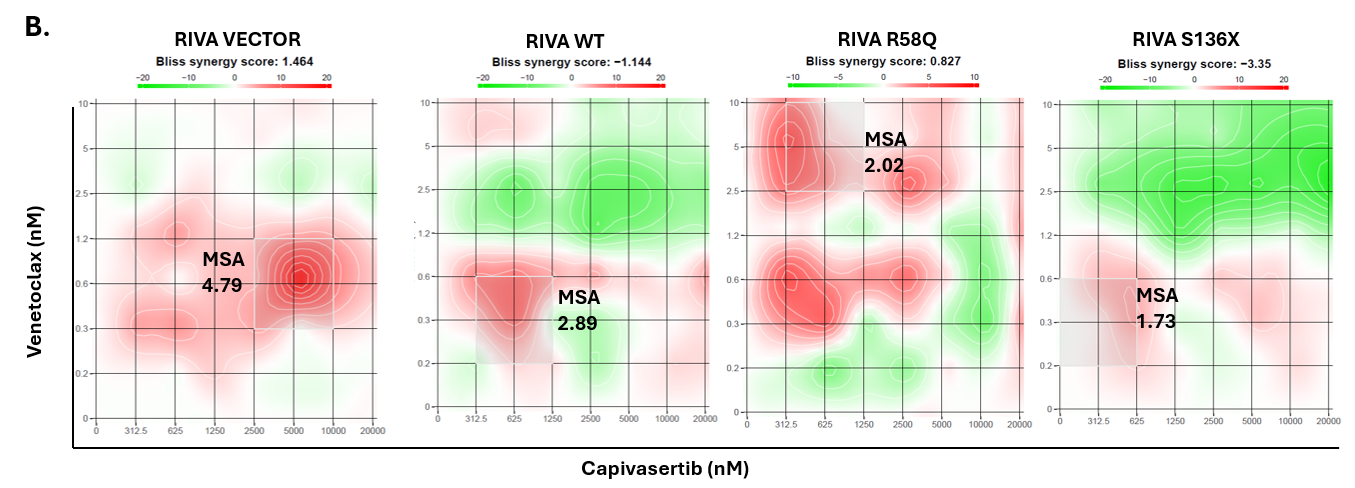


**
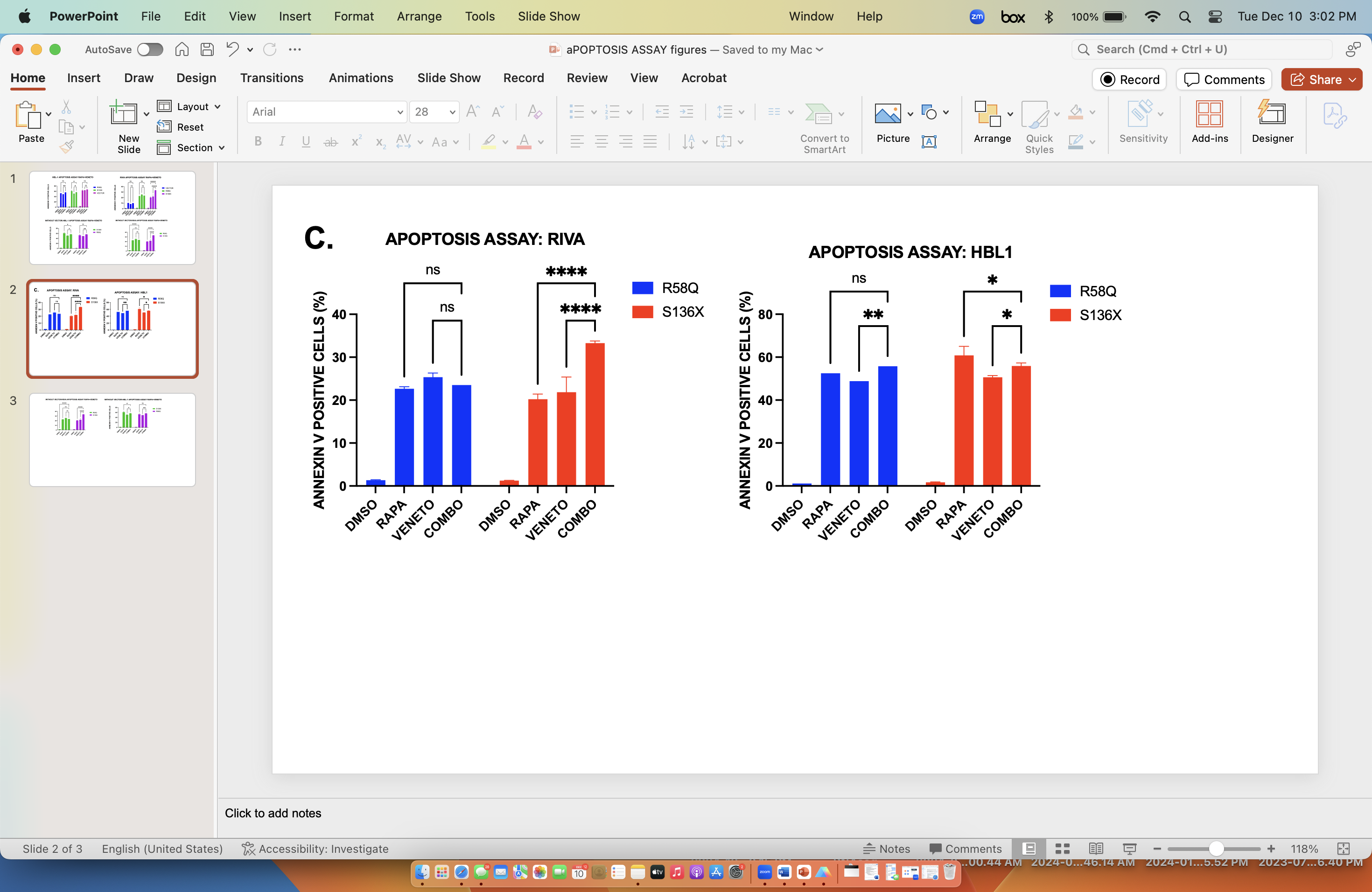
**

**Supplemental Figure 7.** **A.** Synergy of Venetoclax with covalent BTKis in RIVA cells. **B.** Synergy of Venetoclax with Capivasertib in RIVA cells. C. Annexin V apoptosis assay of RIVA and HBL1 cells containing BCL10 R58Q and S136X mutants treated with venetoclax (2nM for RIVA, 5uM for HBL1), rapamycin (2nM) or the combination.


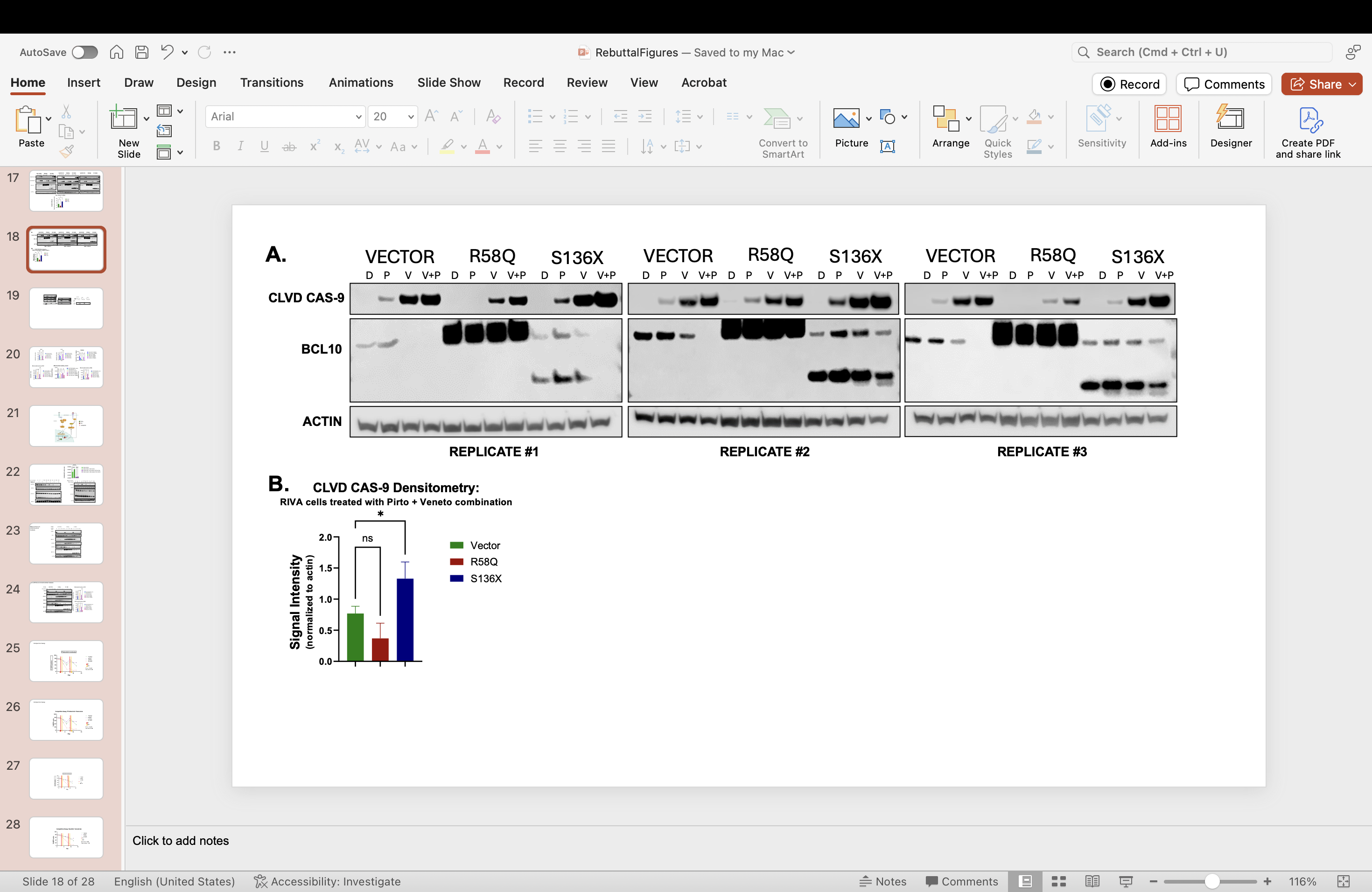


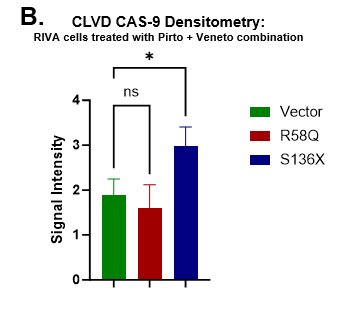


**Supplemental Figure 8.** **Sensitivity to Pirtobrutinib and Venetoclax combination is retained and enhanced in BCL10 mutants. A.** Triplicate western blots of RIVA cells treated with single agent and combination pirtobrutinib and venetoclax. **B.** Densitometry analysis of cleaved caspase-9 in RIVA vector cells compared to BCL10 mutants.

**
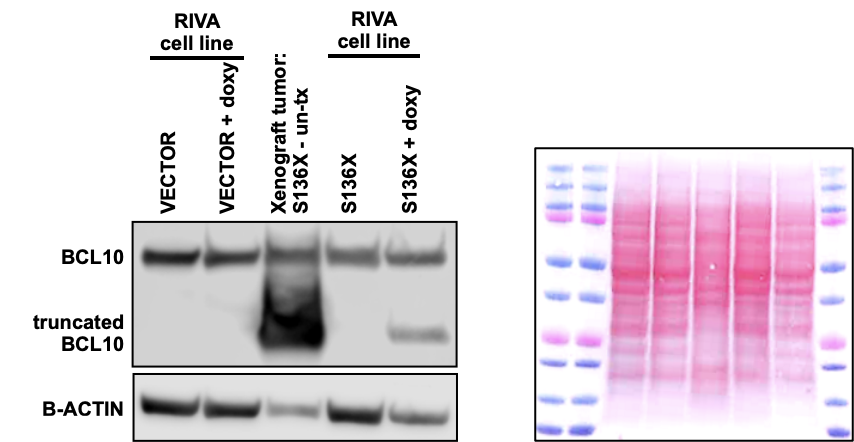
**

**Supplemental Figure 9. RIVA S136X tumor xenograft.** Western blot for cells isolated from control mice with the xenograft tumor. The S136X protein was induced in the mice using mouse food containing doxycycline. RIVA plvx-tetone cells alone were used as a positive control to show the truncated BCL10 by doxycycline induction. RIVA plvx-tetone cells uninduced were used as negative control.

**
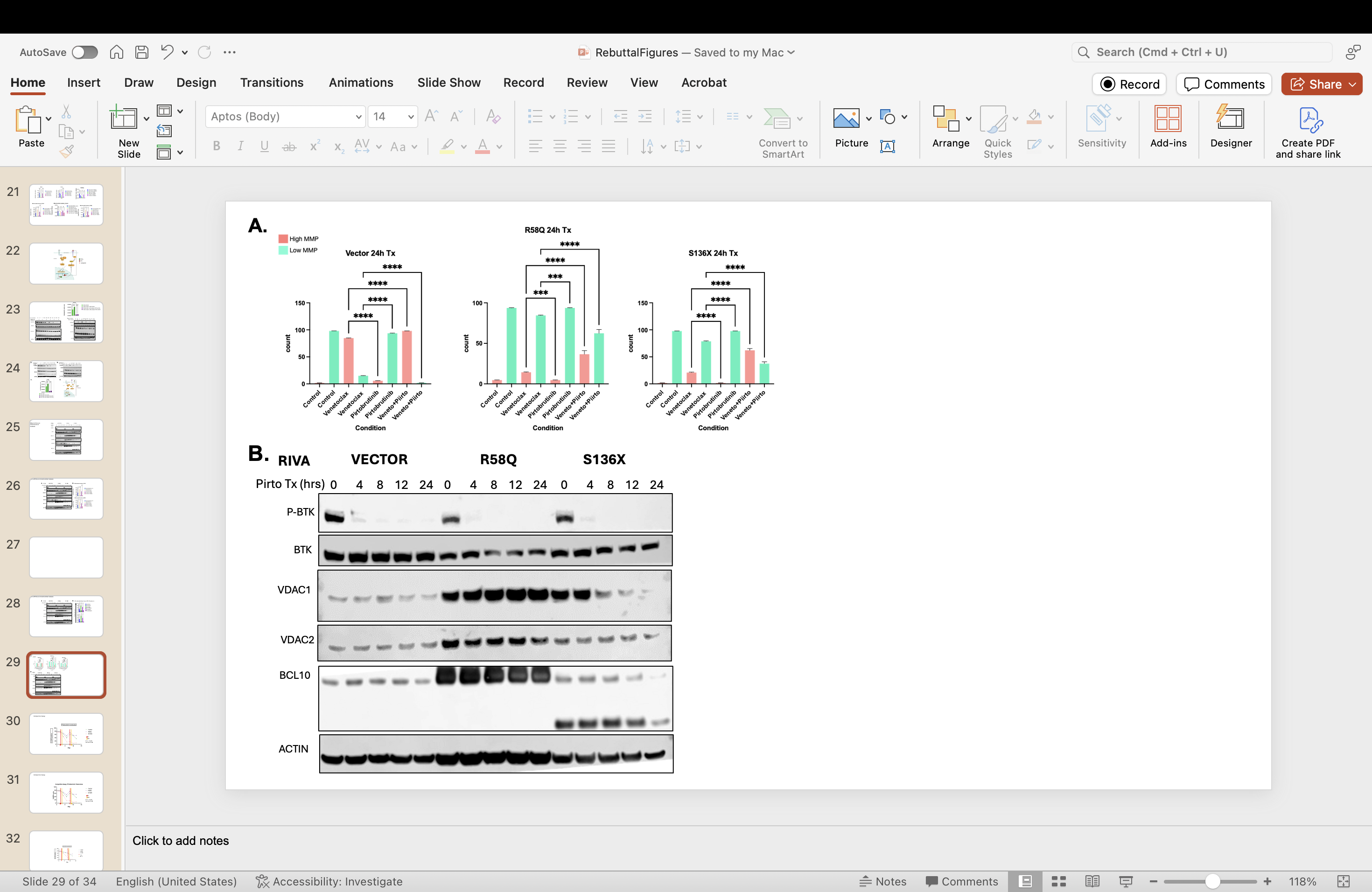
**


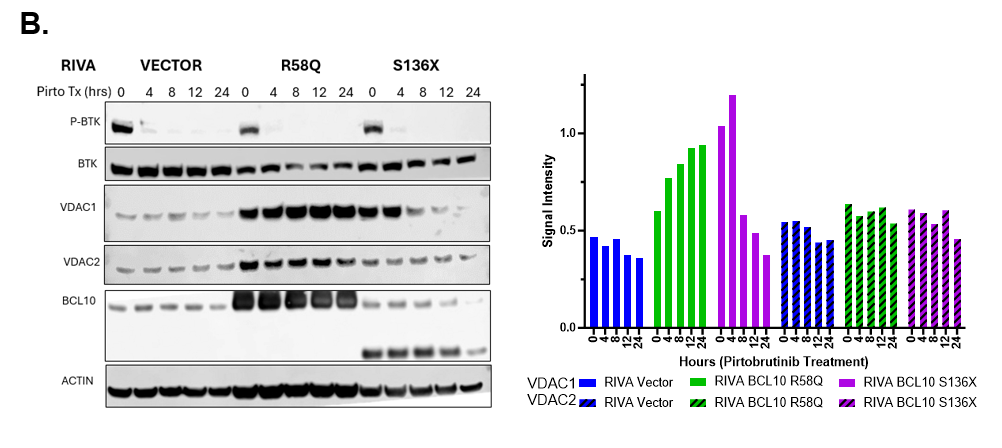


**Supplemental Figure 10. Mitochondrial membrane potential and VDAC and TOMM levels post drug treatment. A.** RIVA cells were treated for 24 hours with control (DMSO), pirtobrutinib, venetoclax or the combination and mitochondrial membrane potential (MMP) was measured using Attune Nxt flow cytometer. The bar shows the average of the shift in the high MMP (orange) to low MMP (green) due to the drug treatment. The shift in the MMP in combination treatment was statistically significant comparing to the venetoclax or pirtobrutinib treatment alone (RIVA Vector, R58Q, S136X; p <0.0001). **B.** Western Blot analysis of RIVA cells treated with 5uM of pirtobrutinib over increasing time points.

**
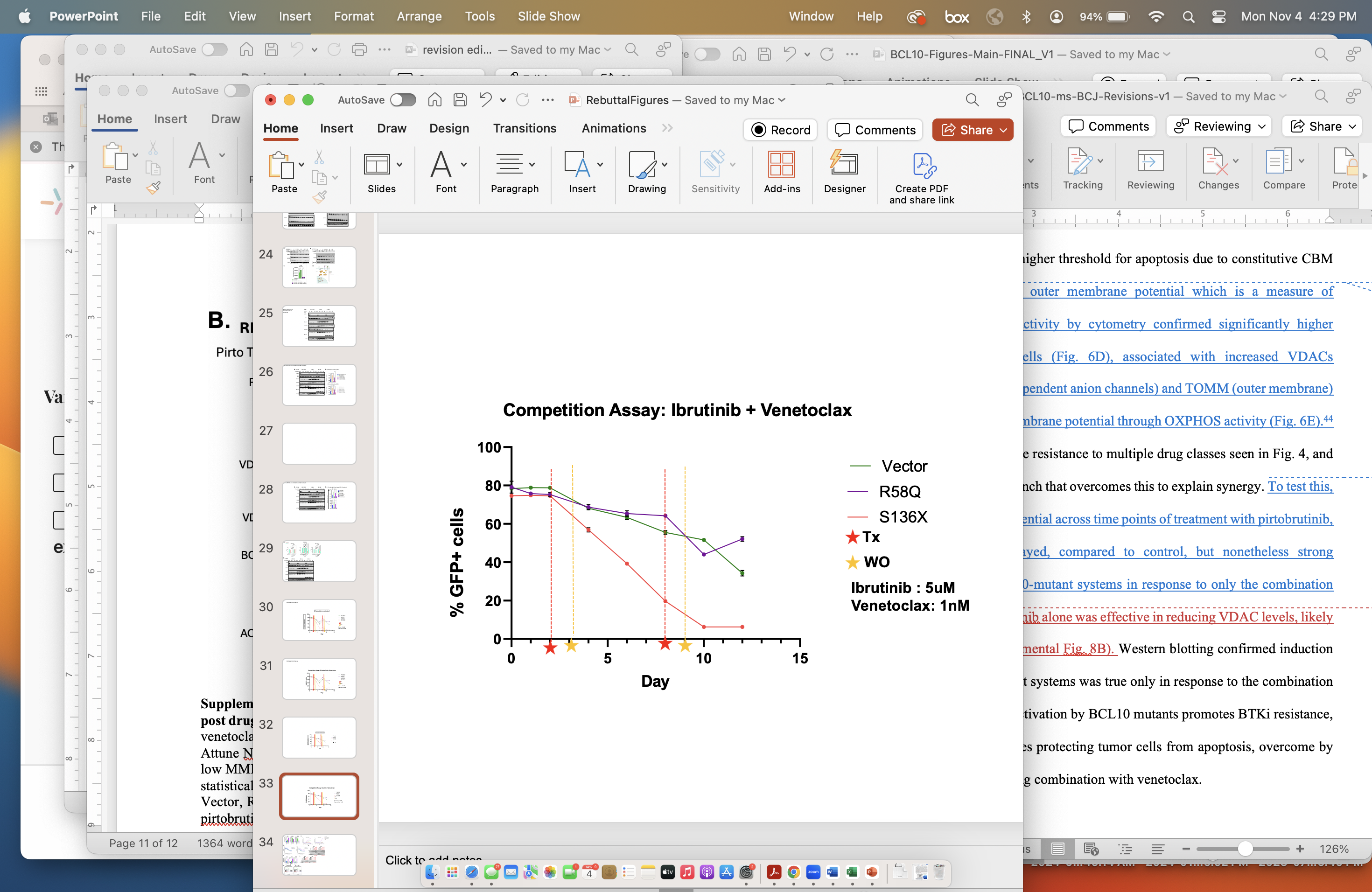
**

**Supplemental Figure 11: Competition Assay of Ibrutinib and Venetoclax in RIVA cells.** RIVA cells containing a stably expressing plvx-GFP construct were treated with the combination of ibrutinib (5uM) and venetoclax (1nM) and GFP expression was monitored using flow cytometry. Cells were treated for 24 hours before being washed out of drug-containing media and allowed to recover for 24 hours before subsequent treatments.

**
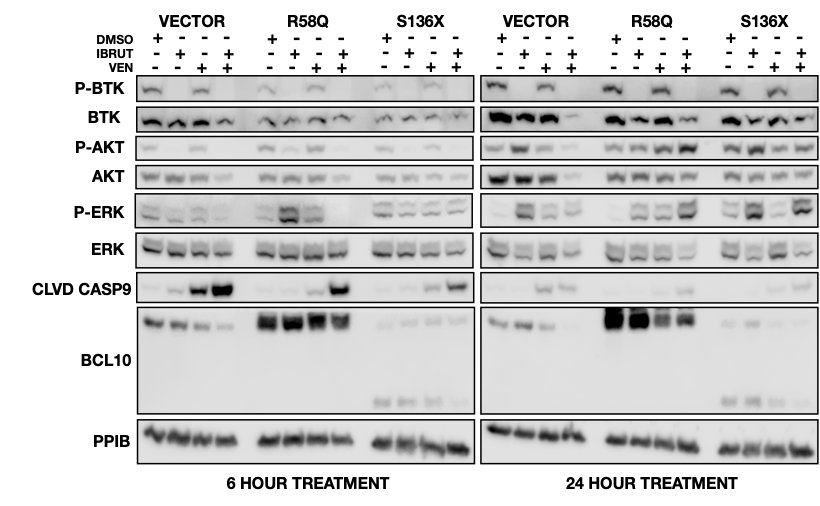
**

**Supplemental Figure 12. Combination therapy of ibrutinib and venetoclax in BCL10 mutant RIVA cells.** Western blot of doxycycline (200ng/ml) induced RIVA cells treated with DMSO, venetoclax, ibrutinib or the comination for 6 and 24-hours.


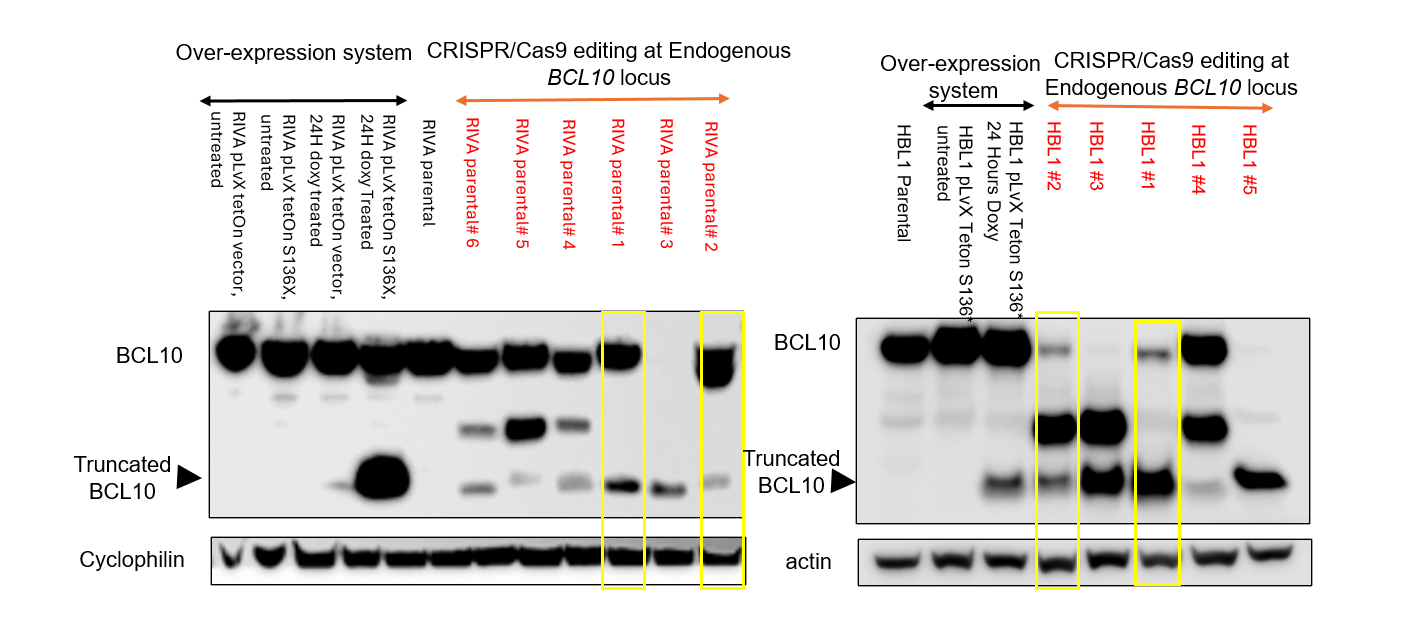


**Supplemental Figure 13. CRISPR/Cas9 editing at endogenous BCL10 locus.** Western blot/screening of clones generated by CRISPR/Cas9 editing to identify heterozygous clones in RIVA and HBL-1 cells for truncated BCL10. Boxed (yellow) clones were used to study drug resistance for BTKi and synergistic drugs combination.
